# Trends and future projections of Parkinson’s disease burden in China: findings from the Global Burden of Disease Study 2023

**DOI:** 10.1016/j.mmr.2026.100077

**Published:** 2026-10-27

**Authors:** Zhe Zhao, Ai-Nan Jia, Wen Ma, Xiao-Hui Xu, Mai-Geng Zhou, Jun-Liang Yuan

**Affiliations:** a Department of Pharmacy, Peking University Third Hospital, Beijing 100191, China; b Institute for Drug Evaluation, Peking University Health Science Center, Beijing 100191, China; c National Center for Chronic and Noncommunicable Disease Control and Prevention, Chinese Center for Disease Control and Prevention, Beijing 100050, China; d School of Basic Medicine, Qingdao University, Qingdao 266071, Shandong, China; e Department of Neurology, Peking University Sixth Hospital, Peking University Institute of Mental Health, NHC Key Laboratory of Mental Health (Peking University), National Clinical Research Center for Mental Disorders (Peking University Sixth Hospital), Beijing 100191, China; f Shandong Center for Disease Control and Prevention, Jinan 250014, China

**Keywords:** Parkinson’s disease (PD), Prevalence, Disability-adjusted life years (DALYs), Burden of disease, Trend

## Abstract

**Background:**

Parkinson’s disease (PD) is a major public health burden in China. We aimed to analyze temporal trends in PD burden across China from 1990 to 2023, and project the future burden through 2050.

**Methods:**

Using data from the 2023 Global Burden of Disease (GBD 2023) database, prevalence, incidence, deaths, disability-adjusted life years (DALYs), years of life lost (YLLs), and years lived with disability (YLDs) of PD in 31 provinces, autonomous regions, municipalities, and the two special administrative regions (SAR) of Hong Kong and Macao of China from 1990 to 2023 were analyzed. Age-standardized rates (ASRs) and the average annual percent change (AAPCs) were calculated. Changes in case numbers were decomposed using a Das Gupta framework, and future burden to 2050 was projected using a probabilistic model-averaging approach.

**Results:**

From 1990 to 2023, the age-standardized prevalence, incidence, and YLD rates of PD increased in China, whereas the age-standardized mortality, YLL, and DALY rates decreased. The number of people living with PD increased from 781.29 thousand (95% UI 629.39–973.58) in 1990 to 3445.69 thousand (95% UI 2804.28–4243.67) in 2023, and incident cases increased from 99.51 thousand (95% UI 82.51–118.00) in 1990 to 379.69 thousand (95% UI 319.28–447.58) in 2023. The age-standardized prevalence rate increased from 106.56 per 100,000 (95% UI 86.16–131.29) to 148.74 per 100,000 (95% UI 122.07–183.52) (AAPC=1.03%, 95% CI 0.98–1.09), whereas the age-standardized mortality, DALY, and YLL rates decreased from 4.69 per 100,000 to 3.78 per 100,000, 92.40 per 100,000 to 77.87 per 100,000, and 77.37 per 100,000 to 56.81 per 100,000, respectively. The age-standardized YLD rate increased from 15.03 per 100,000 to 21.07 per 100,000. Males had consistently higher ASRs than females, although the prevalence rate increased faster in females than in males (AAPC: 1.14% vs. 0.86%, *P<*0.001). In 2023, PD prevalence increased with age, whereas incident cases peaked at ages 70–74 years and deaths at ages 80–84 years. High-middle-Socio-demographic Index (SDI) regions had the highest age-standardized prevalence rate (150.53 per 100,000), while low-SDI regions had the lowest (140.26 per 100,000), with relatively faster increases in low- and low-middle-SDI regions. The highest age-standardized prevalence and incidence rates were observed in Tianjin, Liaoning, Xinjiang, and Hainan. Besides, mortality decreased from 1990 to 2023 in all provinces except Hebei and Jiangsu. In decomposition analyses, changes in age-specific rates contributed most to increases in prevalent and incident cases, whereas population aging contributed most to changes in deaths, DALYs, YLLs, and YLDs. By 2050, the numbers of prevalent cases, incident cases, deaths, and DALYs were projected to reach 6891.38 thousand, 759.37 thousand, 91.66 thousand, and 2528.54 thousand, respectively. In addition, age-standardized prevalence and incidence rates were projected to increase, whereas age-standardized mortality and DALY rates were projected to decrease.

**Conclusions:**

From 1990 to 2023, China experienced a substantial increase in PD burden accompanied by a shift toward a disability-dominated burden, characterized by rising age-standardized prevalence, incidence, and YLDs, but declining mortality and YLLs. Projections through 2050 indicate that population aging will remain the primary driver of future increases in PD burden, underscoring the need for strengthened long-term care, rehabilitation, and region-specific prevention strategies.

## 1 Background

Parkinson’s disease (PD) is the second most common neurodegenerative disease worldwide, pathologically characterized by the progressive loss of dopaminergic neurons in the substantia nigra and the accumulation of Lewy bodies [1], [2], [3]. Clinically, PD manifests as resting tremors, bradykinesia, rigidity, and postural instability, collectively imposing a substantial burden on quality of life and healthcare resources. According to the Global Burden of Disease Study 2021 (GBD 2021), overall measures of PD burden increased substantially over the period from 1990 to 2021, establishing PD as an increasingly prominent global public health concern [4], [5]. The greatest increases were observed among older age groups, suggesting that population aging has substantially contributed to the increasing PD burden [6].

Driven by rapid population aging, PD has imposed a growing social and healthcare burden across China [7]. GBD 2021 data revealed substantial increases in China’s age-standardized PD incidence and prevalence from 1990 to 2021, with both indicators ranking at the top of the G20 and far exceeding global averages [8]. Existing domestic PD epidemiological research is fragmented, and most analyses cover only partial provinces or specific populations. For example, China Health and Retirement Longitudinal Study (CHARLS)-based surveys identified marked regional disparities in PD prevalence [9], and multi-site field surveys reported a standardized PD prevalence of 1.60% among Chinese adults aged 65 and older [10]. Over three decades of rapid socioeconomic development, substantial interprovincial disparities in population and economic conditions have emerged, while nationwide longitudinal evaluations and long-term burden projections for PD remain limited.

Earlier GBD 2021-based studies have laid a basic evidence foundation for assessing PD burden in China. Xu *et al.* [8] assessed national and provincial temporal trends in PD incidence, prevalence, mortality, and DALYs from 1990 to 2021, benchmarked China against G20 nations, and decomposed the contributions of demographic versus disease-specific factors to rising PD cases and deaths. Li *et al.* [5] used a Bayesian age-period-cohort model to project global PD burden through 2046, highlighting that China has among the highest age-standardized PD incidence and prevalence globally. Additional Asia-Pacific research forecasted surging dementia prevalence and predicted East Asia would host the world’s largest PD population by 2050 [2], [11]. However, these studies failed to clarify provincial disparities in China’s fatal and non-fatal PD outcomes, nor did they incorporate Socio-demographic Index (SDI)-stratified provincial comparisons.

Leveraging the updated GBD 2023 database [12], this study aimed to systematically assess temporal trends in six core PD burden metrics [prevalence, incidence, deaths, disability-adjusted life years (DALYs), years of life lost (YLLs), and years lived with disability (YLDs)] across 31 Chinese provinces, Hong Kong, and Macao from 1990 to 2023. We further aimed to characterize variations in PD burden by age, sex, SDI, and geographic region, quantify the contributions of demographic and epidemiological factors to changes in disease burden, and project national trends through 2050.

## 2 Methods

### 2.1 Overview

We analyzed the PD burden in China using data from GBD 2023, covering 31 provinces, autonomous regions, municipalities, and the two special administrative regions of Hong Kong and Macao. The study population comprised the annual population estimates for China from 1990 to 2023. Population denominators varied by calendar year, age, sex, and province. GBD 2023 provides standardized estimates by age, sex, location, and year using multiple data sources and established modelling frameworks. Detailed information on data sources, data processing, and estimation methods has been reported previously [12]. We estimated and compared the prevalence, incidence, deaths, DALYs, YLLs, and YLDs associated with PD by age, sex, SDI, and region from 1990 to 2023, calculated age-standardized rates (ASRs) and average annual percentage changes (AAPCs), and projected trends in PD burden from 2024 to 2050. GBD 2023 used standardized and comparable methods to estimate population demographics, fertility, incidence, and mortality across 204 countries and regions.

### 2.2 Definitions

PD is characterized by both motor and nonmotor manifestations, with the latter including autonomic and neuropsychiatric symptoms. In GBD 2023, PD was identified using ICD-10 codes G20–G20.9 and ICD-11 code 8A00.0, as well as the UK PD Society Brain Bank criteria, physician diagnosis, and use of PD-specific medications. These definitions were used for case ascertainment and cause-of-death mapping rather than clinical severity classification. Although severity-specific health states are incorporated into YLD estimation, individual-level measures such as Hoehn-Yahr stage, MDS-UPDRS scores, and cognitive status are not available. Age-, sex-, SDI-, and region-specific PD data for 1990–2023 were obtained from the publicly available GBD 2023 database [12]. Therefore, institutional review board approval and informed consent were not required.

### 2.3 Measurement indicators

The primary outcomes were PD-related prevalence, incidence, deaths, YLLs, YLDs, and DALYs. Prevalence and incidence represented existing and newly diagnosed PD cases, respectively, while deaths referred to the number of deaths attributable to PD. YLLs quantified years lost due to premature death, YLDs quantified years lived with disability, and DALYs were calculated as the sum of YLLs and YLDs. Estimates were reported with 95% uncertainty intervals (UIs) based on 1000 draws from the GBD framework [13]. Crude rates for each measure were calculated by dividing the corresponding number by the population in that year and were expressed per 100,000 population.

### 2.4 Statistical analysis

ASRs were calculated using direct standardization to the GBD 2023 world standard population and expressed per 100,000. To account for uncertainty in the GBD estimates, the same standardization procedure was applied to each posterior draw, and 95% UIs were defined as the 2.5th and 97.5th percentiles of the resulting distributions.

Temporal trends in PD burden from 1990 to 2023 were assessed using joinpoint regression on log-transformed annual ASRs, following the methodological framework described by Kim *et al*. [14]. Candidate models with zero to three joinpoints were considered, with at least eight annual observations required within each segment. The optimal model was selected using the Bayesian Information Criterion (BIC). For each segment, the estimated annual percentage change (EAPC) was calculated from the slope coefficient on the log scale. The AAPC over the entire study period was calculated as the time-weighted average of the segment-specific EAPCs, with 95% confidence intervals (CIs) estimated using the delta method. Detailed formulas are provided in **Additional file 1: Methods.** Joinpoint regression is an established approach for identifying changes in temporal trends.

Changes in the absolute numbers of PD prevalence, incidence, deaths, DALYs, YLLs, and YLDs between 1990 and 2023 were further examined using a Das Gupta-style decomposition analysis [15]. For each province, age-specific population, case numbers, and rates were used to separate the overall change into three components, including population growth, changes in population age structure, and changes in age-specific rates. The contribution of each component was expressed as a percentage of the corresponding burden in 1990. This decomposition approach separates changes in an aggregate outcome into additive contributions from demographic composition and age-specific rates. Detailed formulas and derivations are provided in **Additional file 1: Methods**.

PD burden from 2024 to 2050 was projected using an ensemble probabilistic model-averaging framework based on annual GBD 2023 time-series data [2], [16], which was fitted to population-level annual time-series data from the 2023 GBD, rather than an individual-level etiological or mortality risk model. Medications, comorbidities, and environmental or behavioral exposures were not included as explicit predictors because consistent longitudinal covariate data were unavailable at the required outcome-specific and geographical resolution. Candidate models included linear and quadratic regression, natural cubic spline regression, piecewise linear regression, autoregressive integrated moving average (ARIMA), and random-walk models, with residual-corrected specifications considered where applicable. Model performance was evaluated by withholding the most recent 11 years of data and comparing projected values with observed estimates using root mean squared error and bias. Candidate models were combined using BIC-based weights, and predictive simulations were used to quantify forecasting uncertainty. Final projections were summarized as mean estimates with 95% uncertainty intervals. Detailed model specifications, validation procedures, weighting methods, and uncertainty estimation are provided in **Additional file 1: Methods**.

Analyses were conducted at the national and provincial levels and were stratified by age, sex, SDI, and geographic region, where applicable. The 31 provinces, Hong Kong, and Macao were classified into 5 SDI groups according to their province-level SDI values in 2023. These fixed 2023 SDI groupings were applied throughout 1990–2023 to allow consistent comparison of temporal trends among regions with different contemporary levels of socioeconomic development.

All data processing, statistical analyses, and visualization were performed using R version 4.5.2. Figures were generated using “ggplot2” and related R packages.

## 3 Results

### 3.1 Overall trends of PD burden in China from 1990 to 2023

From 1990 to 2023, PD burden in China increased steadily and shifted toward a disability-dominated epidemiological profile. The ASRs of prevalence and incidence increased steadily, whereas the ASRs of mortality, YLLs, and DALYs decreased over the same period. By contrast, the ASRs of YLD also increased (Fig. 1; Additional file 1: Fig. S1). The total number of people living with PD rose from 781.29 thousand (95% UI 629.39–973.58) in 1990 to 3445.69 thousand (95% UI 2804.28–4243.67) in 2023. The ASR of prevalence increased from 106.56 per 100,000 (95% UI 86.16–131.29) to 148.74 per 100,000 (95% UI 122.07–183.52), with an AAPC of 1.03% (95% CI 98–1.09) (Table 1; Fig. 1). Similarly, incident PD cases increased from 99.51 thousand (95% UI 82.51–118.00) in 1990 to 379.69 thousand (95% UI 319.28–447.58) in 2023. The age-standardized incidence rate increased from 13.17 per 100,000 (95% UI 11.10–15.57) to 16.26 per 100,000 (95% UI 13.86–18.88), with an AAPC of 0.64% (95% CI 0.61–0.68) (Fig. 1; Additional file 2: Table S1). In contrast, PD-related deaths and age-standardized mortality rates exhibited a divergent pattern. The number of deaths increased from 29.17 thousand (95% UI 25.02–35.14) in 1990 to 82.93 thousand (95% UI 69.15–98.32) in 2023, whereas the age-standardized mortality rate declined from 4.69 per 100,000 (95% UI 4.00–5.67) to 3.78 per 100,000 (95% UI 3.15–4.51), with an AAPC of −0.65% (95% CI −1.11 to −0.19) (Fig. 1; Additional file 2: Table S1). Consistently, the age-standardized DALY rate decreased from 92.40 per 100,000 (95% UI 80.59–108.17) in 1990 to 77.87 per 100,000 (95% UI 67.44–91.16) in 2023, with an AAPC of −0.51% (95% CI −0.84 to −0.19). The age-standardized YLL rate also declined from 77.37 per 100,000 (95% UI 66.55–92.84) to 56.81 per 100,000 (95% UI 48.01–66.93), with an AAPC of −0.96% (95% CI −1.39 to −0.52), indicating a reduction in the age-standardized premature mortality burden. By contrast, the age-standardized YLD rate increased from 15.03 per 100,000 (95% UI 10.50–20.76) in 1990 to 21.07 per 100,000 (95% UI 14.46–29.25) in 2023, with an AAPC of 1.07% (95% CI 1.00–1.13), reflecting an increasing disability burden associated with PD (Additional file 1: Fig. S1; Additional file 2: Table S1). Taken together, these results indicate a structural transition in PD burden in China, with disability increasingly outweighing premature mortality as the dominant component of health loss.

**Fig. 1:**
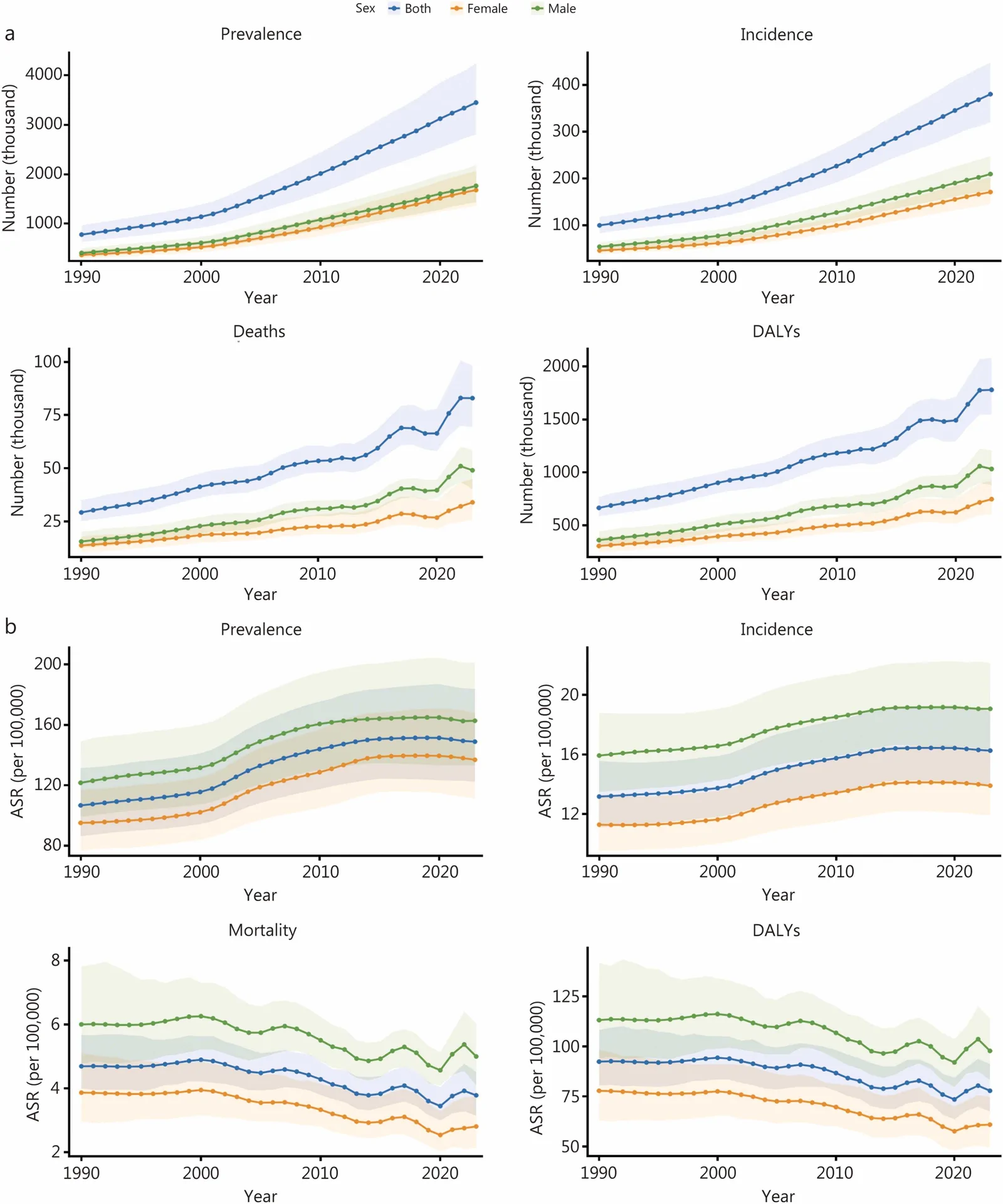
Annual trends in PD prevalence, incidence, deaths, and DALYs by sex in China, 1990–2023. Numbers of prevalence, incidence, mortality, and DALYs (**a**) are expressed in thousands; age-standardized rate (ASR), including prevalence, incidence, deaths, and DALYs (**b**) are expressed per 100,000. Shaded areas denote 95% uncertainty intervals. PD. Parkinson’s disease; DALYs. Disability-adjusted life years.

**Table 1:** Changes in the number of PD patients, age-standardized prevalence rate, and AAPC in China, 1990−2023.

**Characteristics**	**1990**		**2023**		**AAPC [% (95% CI)]**
	**Number of cases (thousand, 95% UI)**	**ASR (per 100,000, 95% UI)**	**Number of cases (thousand, 95% UI)**	**ASR (per 100,000, 95% UI)**	
**Overall**	781.29 (629.39–973.58)	106.56 (86.16–131.29)	3445.69 (2804.28–4243.67)	148.74 (122.07–183.52)	1.03 (0.98−1.09)
Sex					
Female	371.38 (298.43–460.14)	94.95 (76.51–116.86)	1680.79 (1357.73–2063.30)	136.77 (111.06–167.63)	1.14 (1.08−1.20)
Male	409.90 (330.77–506.82)	121.52 (99.06–148.93)	1764.90 (1434.50–2189.44)	162.59 (132.88–201.24)	0.86 (0.80−0.93)
Age group (years)					
20−24	0.29 (0.13–0.54)	0.22 (0.10–0.41)	0.23 (0.11–0.39)	0.32 (0.15–0.55)	1.16 (1.01–1.32)
25−29	1.66 (0.75–3.09)	1.51 (0.68–2.80)	1.73 (0.81–2.92)	2.22 (1.04–3.74)	1.15 (1.01–1.29)
30−34	3.91 (2.12–6.49)	4.41 (2.40–7.33)	6.65 (3.78–10.41)	6.19 (3.52–9.69)	0.98 (0.88–1.08)
35−39	9.24 (6.07–13.19)	10.09 (6.63–14.41)	15.42 (10.14–21.72)	13.04 (8.58–18.37)	0.80 (0.66−0.93)
40−44	13.60 (9.09–20.25)	20.21 (13.51–30.09)	22.18 (15.12–32.09)	24.39 (16.62–35.28)	0.62 (0.39−0.84)
45−49	20.60 (14.82–28.20)	39.80 (28.63–54.48)	45.46 (32.44–62.10)	45.57 (32.52–62.26)	0.53 (0.25−0.80)
50−54	35.87 (26.80–48.00)	74.99 (56.02–100.33)	103.74 (76.65–140.77)	87.62 (64.74–118.90)	0.45 (0.28−0.63)
55−59	63.91 (48.08–83.11)	146.93 (110.53–191.07)	223.96 (167.99–294.42)	189.44 (142.10–249.04)	0.79 (0.68−0.89)
60−64	96.68 (70.96–127.56)	272.71 (200.17–359.80)	307.81 (226.99–408.21)	378.02 (278.76–501.32)	0.92 (0.81−1.03)
65−69	135.80 (102.83–172.08)	495.42 (375.14–627.78)	547.61 (421.04–683.88)	717.59 (551.73–896.15)	1.04 (0.94−1.14)
70−74	151.96 (114.32–199.37)	805.01 (605.63–1056.19)	719.95 (531.01–920.79)	1179.19 (869.73–1508.13)	1.09 (1.02−1.15)
75−79	129.81 (98.17–167.06)	1135.97 (859.13–1462.02)	597.33 (462.93–770.52)	1623.73 (1258.39–2094.50)	1.17 (1.13−1.22)
80−84	78.76 (58.03–101.48)	1410.41 (1039.11–1817.26)	442.41 (341.55–573.50)	1969.39 (1520.40–2552.94)	1.06 (0.97−1.14)
85−89	31.58 (23.32–41.40)	1582.17 (1168.28–2073.90)	272.36 (209.19–349.18)	2183.77 (1677.24–2799.66)	1.09 (0.98−1.21)
90−94	6.39 (4.44–8.97)	1696.29 (1178.08–2381.80)	105.99 (75.82–146.23)	2340.53 (1674.24–3229.05)	1.03 (0.93−1.13)
≥95	1.23 (0.74–1.77)	1872.13 (1135.98–2702.37)	32.85 (20.87–46.81)	2645.97 (1680.96–3769.85)	1.05 (0.91−1.19)
SDI level					
High	84.90 (74.87–97.59)	108.76 (91.03−128.79)	407.46 (359.88–458.68)	147.94 (124.87−173.82)	0.95 (0.87−1.03)
High-middle	156.55 (141.79–174.76)	108.19 (93.66−125.23)	727.28 (652.45–803.42)	150.53 (131.52−171.77)	1.01 (0.95−1.06)
Middle	196.05 (177.61–215.19)	103.32 (89.99−117.96)	853.63 (782.26–939.12)	142.20 (125.32−160.85)	0.99 (0.94−1.05)
Low-middle	269.50 (247.29–295.09)	102.24 (90.11−115.48)	1131.48 (1036.76–1233.73)	146.41 (131.19−164.38)	1.11 (1.05−1.18)
Low	76.48 (68.44–84.64)	97.54 (83.96−112.53)	337.33 (301.77–370.45)	140.26 (123.02−160.03)	1.10 (1.06−1.15)

Values for cases and ASRs are presented with 95% uncertainty intervals (UIs) from GBD 2023 estimates. AAPC was derived from joinpoint regression of log-transformed ASRs as the duration-weighted average change across all fitted trend segments; 95% confidence intervals (CIs) were estimated using the delta method. ASR. Age-standardized rate; AAPC. Average annual percent change; SDI. Socio-demographic Index; UI. Uncertainty interval; CI. Confidence interval

### 3.2 National trends by sex

Sex-stratified analyses for 1990−2023 revealed a consistent increase in absolute PD burden across all core metrics. Throughout the study period, males consistently exhibited higher case numbers and ASRs than females, indicating a greater disease burden (Fig. 1; Additional file 1: Fig. S1). The number of male PD cases increased from 409.90 thousand (95% UI 330.77–506.82) in 1990 to 1764.90 thousand (95% UI 1434.50–2189.44) in 2023, with the age-standardized prevalence increasing from 121.52 per 100,000 (95% UI 99.06–148.93) to 162.59 per 100,000 (95% UI 132.88–201.24). Among females, the number of PD cases increased from 371.38 thousand (95% UI 298.43–460.14) to 1680.79 thousand (95% UI 1357.73–2063.30), and the corresponding age-standardized prevalence increased from 94.95 per 100,000 (95% UI 76.51–116.86) to 136.77 per 100,000 (95% UI 111.06–167.63). Despite the higher overall burden among males, the rate of increase was greater among females, as reflected by a significantly higher AAPC (1.14%, 95% CI 1.08−1.20) compared with males (0.86%, 95% CI 0.80−0.93) (Table 1).

Age-standardized mortality and DALY rates exhibited broadly similar temporal patterns in both sexes, with an overall downward trend accompanied by intermittent fluctuations. After 2010, both rates continued to decline overall, although several short-term rebounds were observed in the late 2010s and early 2020 s. Throughout the study period, males consistently had higher age-standardized mortality and DALY rates than females, indicating a greater burden of PD-related mortality and overall health loss among males (Fig. 1**b**). Similarly, age-standardized YLL rates were persistently higher in males, displaying an overall declining trend with intermittent fluctuations and a gradual rebound in recent years, whereas female age-standardized YLL rates declined more steadily over time (Additional file 1: Fig. S1b). In contrast, age-standardized YLD rates increased progressively in both sexes from 1990 to the mid-to-late 2010s before leveling off in recent years, while remaining consistently higher in males than in females (Additional file 1: Fig. S1b). Overall, these findings indicate a persistent male predominance in the PD burden, alongside a faster expansion of disability-related outcomes among females, underscoring the need for sex-specific prevention and long-term care strategies.

### 3.3 National trends by age groups

From 1990 to 2023, PD burden increased progressively with age, with the largest burden observed among adults aged ≥65 years (Fig. 2). The prevalence rose sharply among individuals aged ≥80 years. In the 85−89-year age group, the number of prevalent cases increased from 31.58 thousand (95% UI 23.32–41.40) in 1990 to 272.36 thousand (95% UI 209.19–349.18) in 2023. Correspondingly, the age-standardized prevalence increased from 1582.17 per 100,000 (95% UI 1168.28–2073.90) to 2183.77 per 100,000 (95% UI 1677.24–2799.66), with an AAPC of 1.09% (95% CI 0.98–1.21) (Table 1). Across age groups, the number of incident cases in 2023 first rose before declining, peaking in the 70−74-year age group, whereas deaths peaked in the 80−84-year age group (Fig. 2). Mortality rates in 2023 increased with age before declining, with the highest rates observed in the 90−94-year age group (Fig. 2). DALY and YLL rates in 2023 peaked among individuals aged 90−94 years before declining (Fig. 2; Additional file 1: Fig. S2), whereas YLD rates rose steadily with increasing age. For both sexes, starting from the 65−69-year age group, YLL burden was consistently approximately 2-fold higher than YLD burden (Additional file 1: Fig. S2).

**Fig. 2:**
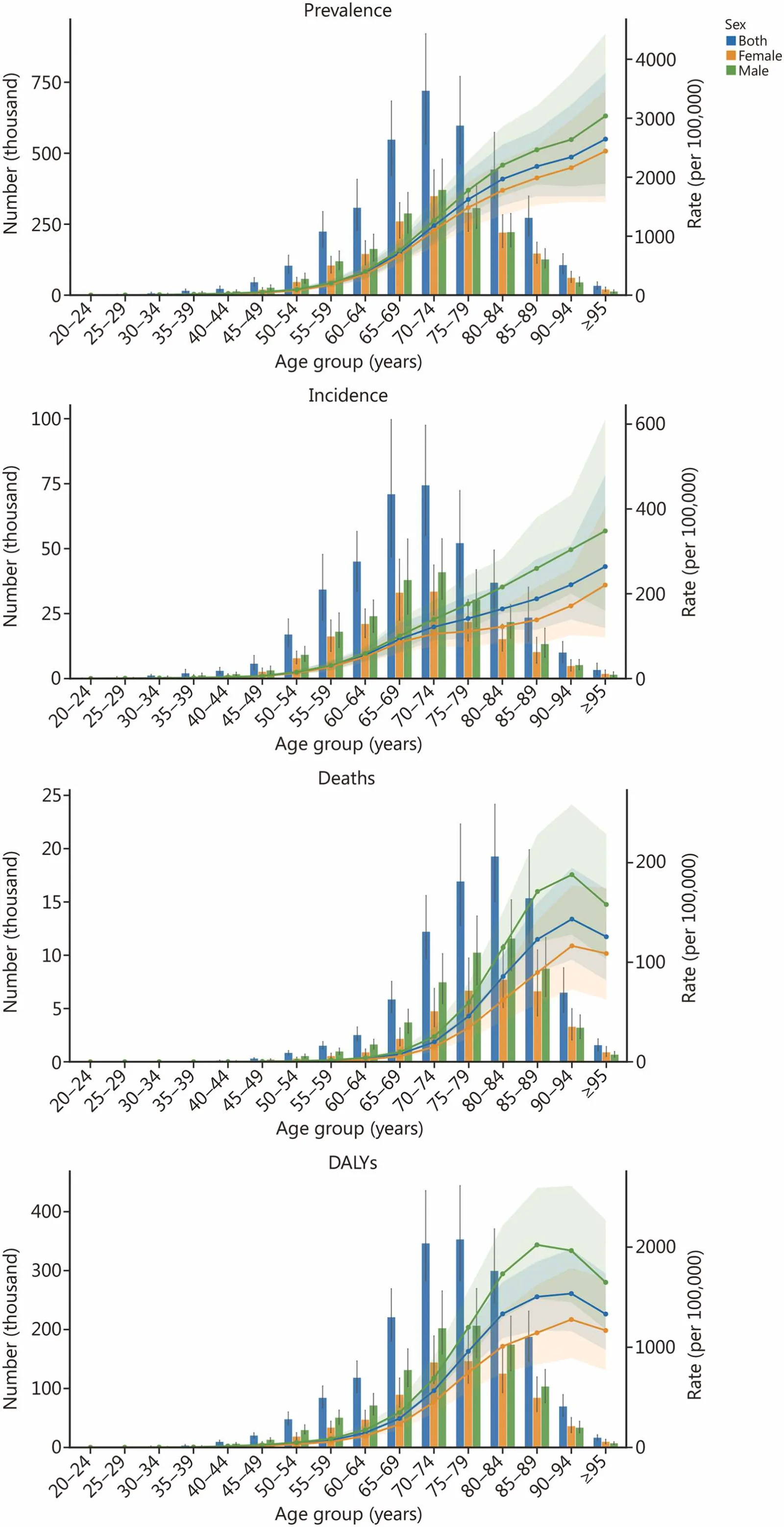
Age-specific burden of PD by sex in China, 2023. Numbers and rates of prevalence, incidence, deaths, and DALYs are shown across age groups. Bars represent absolute numbers (thousand), and lines represent age-specific rates per 100,000 for both sexes combined, females, and males. Error bars and shaded bands indicate 95% uncertainty intervals. PD. Parkinson’s disease; DALYs. Disability-adjusted life years.

Longitudinal analysis of age-standardized indicators from 1990 to 2023 revealed consistent demographic disparities, alongside distinct temporal trends of PD burden across population subgroups. Prevalence, incidence, and disability burden increased over time, whereas death-related measures stabilized or declined, with pronounced age and sex differences. Rates among adults aged ≥85 years were substantially higher than those in younger age groups (Table 1). Elderly males exhibited greater temporal variability and higher peak mortality than females, whose age-specific burden curves were comparatively flatter (Additional file 1: Fig. S3a). AAPC estimates showed positive trends in prevalence and YLD rates across all age groups, with generally larger increases in females than in males, particularly in middle-aged and older age groups. Incidence rates also increased in most age groups, although the magnitude of increase generally attenuated at older ages, with a slight decrease observed in males aged ≥95 years. In contrast, AAPCs for age-specific mortality and YLL rates were negative through the 80–84-year age group but became positive among those aged ≥85 years (Additional file 1: Fig. S3b). Overall, PD burden was concentrated in the older population, with marked age-related differences in temporal trends.

### 3.4 National trends by SDI

We investigated temporal trends in the number and ASRs of PD prevalence, incidence, deaths, DALYs, YLLs, and YLDs across SDI subgroups in China from 1990 to 2023 (Additional file 1: Figs. S4, S5). Among the SDI groups, low-middle SDI regions experienced the largest absolute increase in prevalent PD cases, from 269.50 thousand (95% UI 247.29–295.09) in 1990 to 1131.48 thousand (95% UI 1036.76–1233.73) in 2023. The age-standardized prevalence increased from 102.24 per 100,000 (95% UI 90.11–115.48) to 146.41 per 100,000 (95% UI 131.19–164.38), with the highest AAPC among the SDI groups (1.11%, 95% CI 1.05–1.18) (Table 1; Additional file 1: Fig. S4a).

In 2023, high-middle SDI regions exhibited the highest age-standardized prevalence of PD at 150.53 per 100,000 (95% UI 131.52–171.77), whereas low-SDI regions showed the lowest age-standardized prevalence at 140.26 per 100,000 (95% UI 123.02–160.03) (Table 1; Additional file 1: Fig. S4b). Despite this lower baseline level, low-SDI regions experienced a remarkable increase in age-standardized prevalence during 1990−2023 (AAPC=1.10%, 95% CI 1.06−1.15) (Table 1; Additional file 1: Fig. S4b).

For incidence, the age-standardized incidence rate increased more rapidly in low-middle SDI regions (AAPC=0.73%, 95% CI 0.67–0.79) than in high-SDI regions (AAPC=0.57%, 95% CI 0.53–0.61) (Additional file 1: Fig. S4a-b; Additional file 2: Table S1). From 1990 to 2023, age-standardized mortality, DALYs, and YLLs rates declined across all SDI groups, with the most pronounced decreases observed in middle-SDI regions (AAPCs of −0.94%, −0.76%, and −1.15%, respectively) (Additional file 1: Fig. S4a-b, Fig. S5a-b; Additional file 2: Table S1). In contrast, age-standardized YLD rates increased in all SDI strata, with the steepest increase occurring in the low-SDI regions (AAPC=1.16%, 95% CI 1.07–1.25) (Additional file 1: Fig. S5a-b; Additional file 2: Table S1).

From 1990 to 2023, males consistently had higher age-standardized prevalence than females across all SDI strata. Regional disparities were more pronounced among males, whereas female rates were relatively consistent across SDI groups (Additional file 1: Fig. S6a). AAPC analyses revealed two broad patterns by SDI and sex: prevalence, incidence, and YLDs increased in both sexes across all SDI groups, with higher AAPCs among females than males. Conversely, age-standardized mortality, DALY, and YLL rates declined across all groups, with more rapid reductions in middle- and high-SDI regions and among females (Additional file 1: Fig. S6b).

### 3.5 Provincial trends

To further characterize the spatial heterogeneity of PD burden across China, we examined provincial-level variations in key burden indicators from 1990 to 2023. ASRs of prevalence, incidence, and YLDs increased significantly in different provinces during the study period (Additional file 2: Table S2). By 2023, the highest ASRs of prevalence and incidence were observed in Tianjin, Liaoning, Xinjiang, and Hainan (Additional file 2: Table S2). For both indicators, Macao exhibited the highest AAPC (1.30% for prevalence and 1.08% for incidence), followed by Qinghai, whereas Shaanxi showed the lowest AAPC (Additional file 1: Fig. S7; Additional file 2: Table S2).

Compared with 1990 levels, age-standardized PD mortality had decreased substantially in most Chinese provinces by 2023, with the only exceptions being Hebei, Jiangsu, and Xizang provinces (Additional file 2: Table S2). In 2023, age-standardized PD mortality was highest in Shanghai, Hebei, Zhejiang, and Liaoning, and lowest in Qinghai, Hubei, and Hainan. The highest AAPCs for age-standardized PD mortality were found in Hebei, Jiangsu, and Xizang, whereas the lowest were observed in Hubei and Sichuan (Additional file 1: Fig. S7). Age-standardized DALY rates declined at the national level, with higher levels observed in Xinjiang, Liaoning, and Hebei and lower levels in Hubei, Hainan, and Qinghai, showing a general concentration of higher burden in northern regions (Additional file 2: Table S2). Hebei, Tianjin, Xizang, and Jiangsu exhibited the highest AAPCs in age-standardized DALY rates, whereas Hubei and Jilin exhibited the lowest AAPCs (Additional file 1: Fig. S7). Age-standardized YLL rates declined overall (Additional file 2: Table S2), with the highest AAPC observed in Hebei and the lowest in Sichuan (Additional file 1: Fig. S7). In contrast, age-standardized YLD rates increased steadily nationwide (Additional file 2: Table S2). Macao exhibited the highest AAPC, whereas Shaanxi and Shanxi showed the lowest (Additional file 1: Fig. S7). Collectively, these findings indicate a sustained increase in PD burden over the past three decades, accompanied by marked and widening provincial heterogeneity.

### 3.6 Decomposition of driving factors

To identify the principal drivers of provincial variation in PD burden, we conducted a decomposition analysis to examine changes in prevalence, incidence, and DALYs from 1990 to 2023. Population aging exerted a consistently positive effect on PD burden across various provinces, whereas population growth contributed negatively to PD burden in Sichuan (Fig. 3). Increases in age-specific rates were the primary drivers of growth in prevalent and incident cases nationwide. For prevalent cases, the strongest contributions were observed in Chongqing, Ningxia, and Beijing, while comparatively weaker effects were seen in Sichuan, Xizang, and Shanxi (Fig. 3). Population aging emerged as the dominant contributor to PD-related deaths, DALYs, YLLs, and YLDs across China. The contribution of population aging was particularly large in Chongqing and comparatively small in Xizang (Fig. 3; Additional file 1: Fig. S8). Age-specific mortality rates contributed positively to PD mortality only in Jiangsu, whereas an increase in age-specific DALY rates was observed in Jiangsu, Hebei, and Xizang (Fig. 3; Additional file 1: Fig. S8). Overall, population aging has been the dominant driver of the growing PD burden across China.

**Fig. 3:**
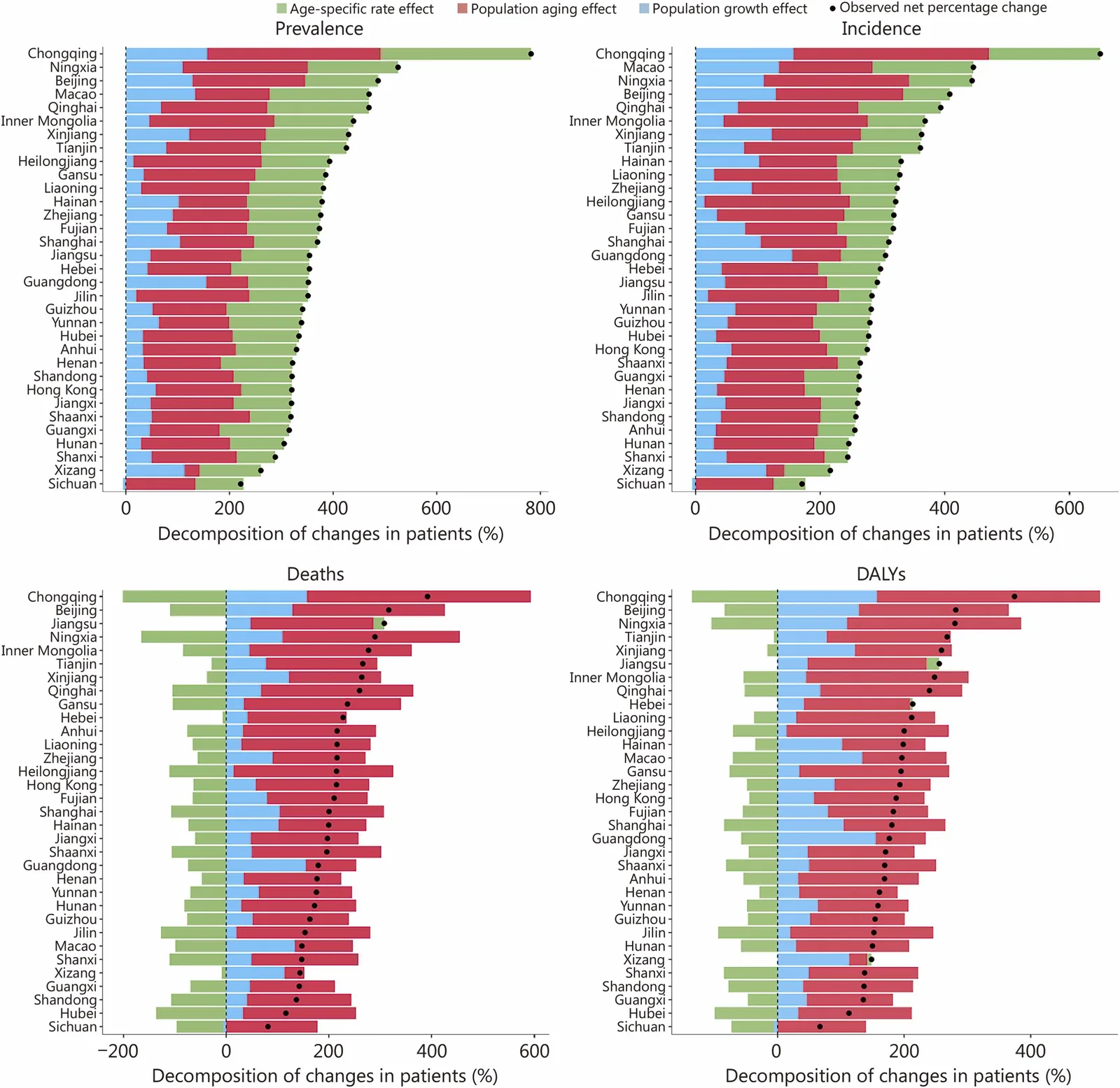
Decomposition of changes in PD burden across Chinese provincial-level regions, 1990–2023. Decomposition of changes in the numbers of prevalence, incidence, deaths, and disability-adjusted life years (DALYs). Stacked bars show the contributions of population growth, population aging, and changes in age-specific rates to the overall change in each burden measure, expressed as percentages of the corresponding 1990 values. Positive values indicate contributions to an increase in burden, whereas negative values indicate contributions to a decrease. Black dots indicate the observed net percentage change in each burden measure between 1990 and 2023, corresponding to the sum of the three decomposition components. PD. Parkinson’s disease; DALYs. Disability-adjusted life years.

### 3.7 Future projections of PD burden

We projected future trends of PD prevalence, incidence, deaths, DALYs, YLLs, and YLDs across China for the period 2024−2050. The total number of people living with PD in China is projected to reach 6891.38 thousand by 2050 (Fig. 4**a**). Incident PD cases are projected to increase to 538.40 thousand in 2030 and 759.37 thousand in 2050 (Fig. 4**a**), whereas PD-related deaths are expected to be 80.20 thousand in 2030, 85.58 thousand in 2040, and 91.66 thousand in 2050 (Fig. 4**a**). The total DALYs attributable to PD are projected to increase to 1790.65 thousand in 2030, 2128.83 thousand in 2040, and 2528.54 thousand in 2050 (Fig. 4**a**). Over the same period, age-standardized prevalence and incidence rates were predicted to continue increasing, whereas age-standardized mortality and DALY rates were expected to decline (Fig. 4**b**). The absolute number of PD-related YLLs is projected to remain relatively stable, with only a slight increase through 2050, whereas YLDs are expected to increase until approximately 2040 before plateauing at higher levels. In contrast, the age-standardized YLL rate is forecast to decline steadily, whereas the YLD rate is expected to increase until the mid-2040s and then level off (Additional file 1: Fig. S9a-b). Collectively, these projections indicate that although the mortality-related burden may stabilize or decline, the overall PD burden in China will continue to grow substantially by 2050, driven primarily by an expanding population living with long-term disability.

**Fig. 4:**
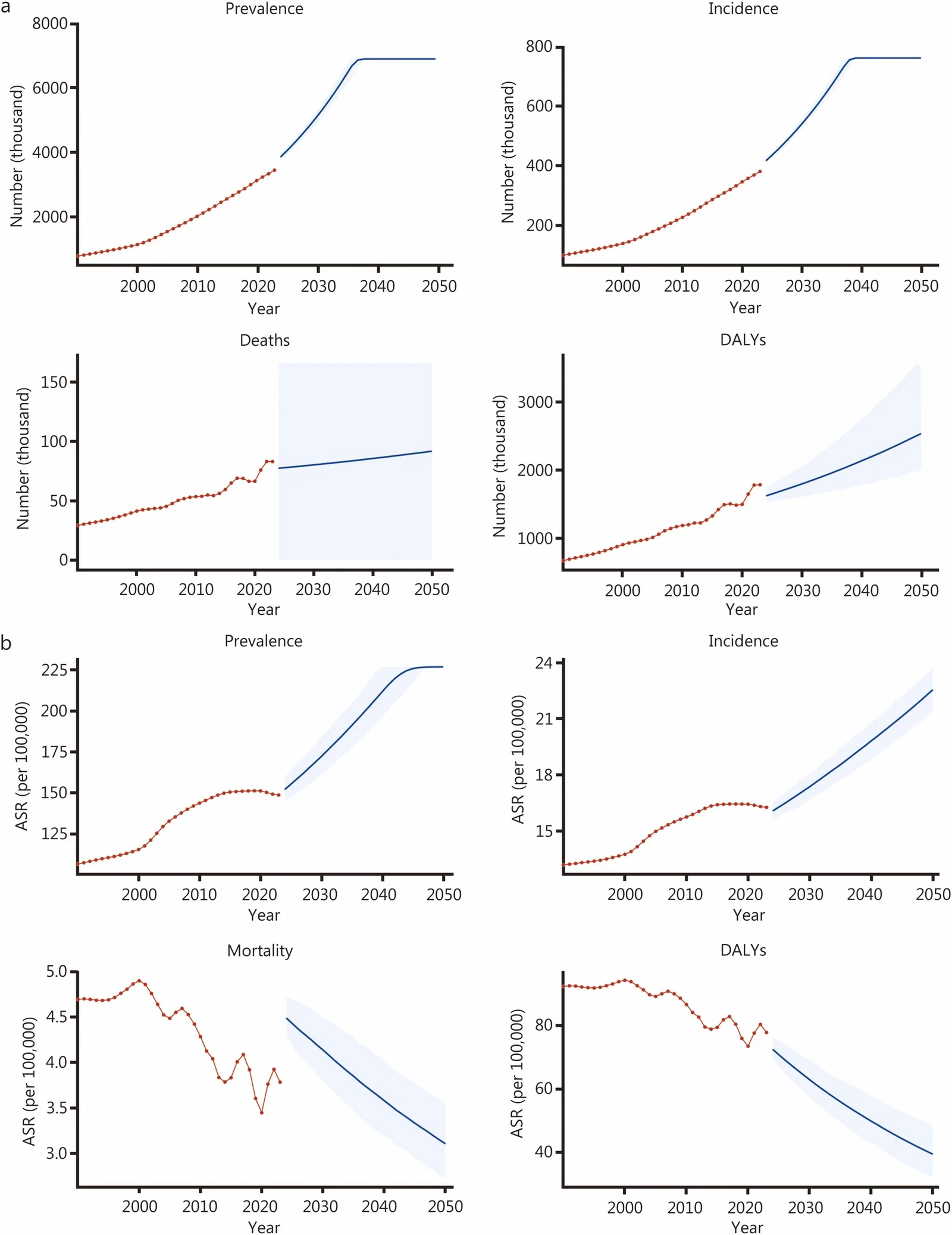
Projected trends in PD burden in China, 2024–2050. Numbers of prevalent cases, incidence, deaths, and DALYs (**a**), and corresponding age-standardized rates (ASRs) of prevalence, incidence, mortality, and DALYs (**b**). Red lines indicate observed estimates for 1990–2023 and blue lines indicate projections for 2024–2050. Shaded areas represent 95% uncertainty intervals. Projections were derived using an ensemble probabilistic model-averaging framework. PD. Parkinson’s disease; DALYs. Disability-adjusted life years.

## 4 Discussion

Using GBD 2023 estimates, this study assessed temporal, demographic, and geographic patterns of PD burden in China from 1990 to 2023 and projected trends through 2050. Consistent with previous GBD-based studies, PD burden was concentrated among older adults and males, with substantial provincial heterogeneity [5], [8]. Our findings further showed increasing prevalence, incidence, and disability burden, alongside declining age-standardized mortality and YLLs. Compared with previous studies, this analysis provides updated estimates through 2023, broader province-level decomposition, and SDI comparisons across six burden measures, and China-specific projections through 2050.

The number of people living with PD in China has increased substantially over the past three decades, alongside a distinct structural transition in disease burden, characterized by sustained increases in age-standardized prevalence, incidence, and YLDs, but concurrent declines in age-standardized mortality, YLLs, and DALYs. Decomposition analyses indicated that increasing age-specific rates were the principal drivers of growth in prevalent and incident cases, whereas population aging contributed most to increases in deaths and health-loss measures. The divergent trajectories of PD burden indicators from 1990 to 2023 indicate PD in China has increasingly become a chronic, disabling condition rather than a predominantly fatal disorder.

This shifting pattern likely stems from multiple interrelated factors. First, enhanced diagnostic capacity, expanded access to neurology services, and greater public awareness have reduced underdiagnosis and delayed identification, especially at earlier disease stages [17], [18], leading to higher recorded incidence and prevalence. Second, widespread access to and standardization of symptomatic therapies (e.g., levodopa-centered therapy, adjunct pharmacologic strategies, and in selected settings, deep brain stimulation) have likely reduced PD-related mortality and prolonged survival, thereby extending the duration of disability and raising cumulative YLD burden [19], [20], [21], [22]. Third, accelerated population aging has expanded the population at high risk, amplifying both disease onset and the number of individuals living with functional impairment [23]. Notably, the declining DALY rate should not be interpreted as a diminished societal impact, rather, it reflects a shift of health burden from premature mortality (YLLs) to prolonged disability (YLDs).

This shift carries substantial implications for China’s health and social care systems. As patients live longer with progressive motor and non-motor symptoms, unmet needs will increasingly center on rehabilitation, long-term care, community-based management, and prevention of secondary complications such as falls, aspiration pneumonia, malnutrition, and depression. Furthermore, the sustained increase in age-standardized incidence suggests that drivers beyond population structure, including potential environmental exposure and lifestyle-related risks, may contribute to disease occurrence, underscoring the need for strengthened etiological research and targeted risk factor surveillance in China. Overall, although the mortality risk associated with PD in China has been substantially mitigated, the long-term disability burden continues to intensify, and the overall PD burden will rise further with ongoing economic development and accelerating population aging.

Our analyses demonstrated consistent male predominance in PD burden, with males consistently exhibiting higher absolute case counts and ASRs across all core measures. This pattern aligns with global epidemiological evidence and likely reflects a combination of biological susceptibility, differential occupational and environmental exposures, and sex-specific behavioral and healthcare-seeking factors [2]. The proposed biological mechanisms include the modulatory role of sex hormones in dopaminergic pathways and neuroinflammation, as well as potential sex-linked genetic vulnerability [24], [25]. Non-biological contributors may include higher exposure to environmental toxins and a greater prevalence of cardiovascular and metabolic comorbidities among men [26]. Despite the higher overall burden in males, the growth trajectory of disability-related outcomes was steeper in females. Longer life expectancy among women may increase survival into advanced age groups, where PD prevalence and functional impairment are most pronounced. Historical under-recognition of PD in women may also have contributed to the observed sex-specific pattern [27]. The faster expansion of the disability burden among females highlights that health systems should anticipate growing demand for sex-responsive long-term care, fall prevention programs, and caregiver support tailored to the aging female population.

Age-stratified analyses demonstrated that age is the strongest determinant of PD burden in China. Across all indicators, rates increased sharply after midlife and reached their highest levels in the oldest age groups, with the most pronounced growth observed among individuals aged ≥95 years. This steep age gradient matches the progressive neurodegenerative nature of PD, cumulative neuronal vulnerability, multimorbidity, and declining physiological reserves all compound with advancing age. Clinically, older patients more frequently experience severe motor and non-motor symptoms and face a higher risk of complications, including aspiration pneumonia, falls, and malnutrition, all of which increase disability and healthcare utilization [28], [29]. From a population health perspective, this pattern underscores that China’s ongoing aging will remain the principal driver of future PD-related disabilities. Sex and SDI exert secondary modulating effects and contribute to sex‑specific disparities and regional heterogeneity of PD burden across China. However, these variables were only used for stratification rather than multivariable modelling, so formal statistical ranking of their independent contributions is not feasible in the present study.

PD burden varies significantly across regions, both at different SDI levels and across provinces. At different SDI levels, absolute PD prevalence, incidence, deaths, and DALYs increased, mainly driven by population growth and aging intensification. Low and low-middle SDI regions have seen particularly rapid growth of absolute PD burden, reflecting accelerated aging in populations with limited long-term management capacity [6], [30]. High-SDI regions achieve higher case detection and longer survival via better care. In contrast, ASRs grow more slowly in high/high-middle SDI regions. These findings suggest widening disparities in PD burden linked to regional development. Although the use of fixed 2023 SDI quintiles facilitated longitudinal comparisons, it may have attenuated the observed pace of transition in provinces whose socioeconomic position changed substantially during 1990−2023. Environmental and behavioral factors (e.g., air pollution, pesticides, certain metals, and lifestyle-related factors) may also contribute to regional differences in PD burden [31], [32], [33], [34]. However, GBD 2023 does not provide quantitative attributable burden estimates for these factors, so their effects remain speculative.

Beyond SDI stratification, pronounced provincial heterogeneity is observed across all PD burden measures, driven jointly by demographic structure, healthcare capacity, environmental exposure, and disease surveillance systems, with population aging as the leading contributor to regional differences in PD prevalence and incidence. Specifically, provinces with more advanced aging and rapidly growing older populations (e.g., eastern coastal areas and Xinjiang) face higher PD burden [7], while younger, slower-aging provinces (e.g., Shaanxi) have lower growth of PD burden indicators. Disparities in healthcare resources and long-term disease management further shape these spatial patterns [35], [36]. Economically developed provinces (e.g., Shanghai and Tianjin) have lower underdiagnosis (thus raising reported prevalence and incidence) and lower mortality and YLL rates via better access to care [10], while the paradoxically high age-standardized mortality in highly developed provinces stems from more complete reporting, larger oldest-old populations, and more patients surviving to advanced disease stages where complications raise mortality. By contrast, provinces with limited care access and weaker continuity of care (e.g., Hebei, Jiangsu) show slower declines in mortality and health loss indicators, indicating gaps in referral, complication prevention, and long-term follow-up [8]. Northern and industrialized provinces (e.g., Hebei, Liaoning, and Xinjiang) may face higher exposure to industrial pollutants and agricultural chemicals (heavy metals, pesticides) linked to dopaminergic neurotoxicity and elevated PD risk [37], while inter-regional differences in diet, physical activity, and health behaviors may further modify PD susceptibility. Finally, uneven development of provincial surveillance and screening also shapes observed trends. For example, regions that recently expanded neurological disease screening and case-finding (e.g., Macao, Qinghai) have higher AAPCs in prevalence and incidence, reflecting improved diagnostic coverage rather than rising underlying risk [8], [38], while lower AAPCs in other provinces often stem from persistent under-recognition of mild or early PD at the primary care level. Overall, provincial PD burdens are widening over time, driven by the combined effects of the above factors. This heterogeneity suggests that national strategies may benefit from greater adaptation to provincial demographic, healthcare, and epidemiological contexts.

Our study projections for 2024−2050 show PD prevalence, incidence, total case numbers, and YLDs will continue to rise, while the ASRs of mortality, DALYs, and YLLs will decline gradually, indicating further expansion of a disability-dominated burden. While absolute PD-related deaths are projected to see a modest increase, this growth is proportionally smaller than the rise in prevalence, reflecting improved survival and longer disease duration rather than elevated age-specific mortality. Population aging will remain the principal driver of growing absolute case counts, as the proportion of older adults continues to rise. Concurrently, expanded diagnostic coverage (i.e., wider neuroimaging use, stronger primary care capacity, and greater public awareness) will improve early-stage disease detection, pushing up measured prevalence and incidence [12], [35], [36]. Clinical management advances (e.g., optimized pharmacotherapy, deep brain stimulation, and emerging regenerative and rehabilitation approaches) will further reduce life-threatening complications and sustain the decline in mortality and YLLs [39], [40], [41]. However, since current treatments are largely symptomatic rather than curative, longer survival will drive an accumulation of people living with PD-related disabilities, and ongoing industrialization and lifestyle-related exposures will also continue to increase underlying PD risk.

Based on these findings, several policy implications merit further emphasis. First, strengthen earlier recognition of PD among adults aged ≥65 years. Second, expand diagnostic and neurological services in rapidly aging low/low-middle SDI regions. Third, establish surveillance for potentially modifiable environmental and occupational PD risks. Given the dominant contribution of population aging, future strategies should not only focus on reducing avoidable risk but also prioritize delaying disability and strengthening rehabilitation, long-term care, and caregiver support for people living with PD. High-prevalence provinces should strengthen early detection, outpatient care, and rehabilitation to reduce disability, whereas provinces with high mortality and DALY burden need targeted improvements in diagnosis, specialist referral, and prevention of complications such as falls and aspiration.

Several limitations of this study should be noted. First, this study relied on aggregated population‑level data from the GBD 2023 database, which lacks individual clinical information, such as PD disease severity and comorbid dementia status, limiting the performance of relevant subgroup stratified analyses. Furthermore, our death outputs represent deaths attributable to PD, and we did not conduct separate analyses for death certificates with co-recorded PD and dementia diagnoses. Second, our projection model extrapolates historical population-level time-series trends without explicitly including individual behaviors, environmental exposures, comorbidities, or medication-related predictors. This may limit its ability to capture future changes in PD risk and reduce generalizability to other regions. Third, individual genetic data are unavailable in the GBD database, so the potential influence of genetic factors on provincial PD burden heterogeneity cannot be evaluated. Meanwhile, geographical‑provincial disparities observed in PD burden cannot be attributed to location itself, as provincial-level aggregated GBD data cannot disentangle multiple co‑occurring regional contextual factors including socioeconomic status, diagnostic practice, healthcare accessibility, and diverse environmental exposures. Finally, the findings of this study can only provide epidemiological evidence for regional health resource allocation and policy formulation, rather than direct clinical diagnosis, treatment, and prevention criteria for PD. Despite these limitations, this study adopts updated GBD data and a standardized spatiotemporal prediction framework, providing reliable evidence for understanding the temporal and spatial trends of PD burden in China. The analytical workflow also offers a referential paradigm for future chronic neurodegenerative disease burden research. Further integrated studies combining clinical cohorts and multi-omics data will help complement and optimize the targeted prevention and control strategies for PD.

## 5 Conclusions

From 1990 to 2023, the burden of PD in China increased substantially and shifted toward disability-dominated health loss, with rising age-standardized prevalence, incidence, and YLDs, but declining mortality-related and YLL-related rates. Marked heterogeneity across sex, age, SDI, and provinces reflects the unequal effects of population aging and healthcare capacity. Decomposition and projections for 2050 indicate that population aging will remain the primary driver of future growth, underscoring the need to strengthen long-term care, rehabilitation, and region-specific prevention strategies.

## Abbreviations

AAPC: Average annual percent change
YLDs: Years lived with disability
YLLs: Years of life lost
DALYs: Disability-adjusted life years
SDI: Socio-demographic Index
UI: Uncertainty interval
ASRs: Age-standardized rates
BIC: Bayesian Information Criterion
EAPC: Estimated annual percentage change
ARIMA: Autoregressive integrated moving average
RMSE: Root mean squared error

## Funding

This study was supported by the National Natural Science Foundation of China (82404597, 82071552, 22376006, U23A20167), the Chinese Academy of Sciences Grant (JCTD-2021-06), Peking University Clinical Scientist Program (BMU2023PYJH018), Key Clinical Projects of Peking University Third Hospital (BYSY2022053), Open Research Project in State Key Laboratory of Vascular Homeostasis and Remodeling (Peking University, 2025-VHR-O-SY-20), Clinical Medicine Plus X (Young Scholars Project of Peking University, PKU2023LCXQ015), Peking University Medicine Sailing Program for Young Scholars’ Scientific & Technological Innovation, and the Fundamental Research Funds for the Central Universities (BMU2024YFJHPY003), and the Chinese Pharmaceutical Association Hospital Pharmacy Department (CPA-Z05-ZC-2024002).

## Data Availability

The datasets generated during the current study will be available from the corresponding author upon reasonable request.

## Footnotes

Appendix A Supplementary data associated with this article can be found in the online version at doi:10.1016/j.mmr.2026.100077.

## References

[1] Ben-Shlomo Y., Darweesh S., Llibre-Guerra J., Marras C., San Luciano M., Tanner C. (2024). The epidemiology of Parkinson’s disease. Lancet.

[2] Su D., Cui Y., He C., Yin P., Bai R., Zhu J. (2025). Projections for prevalence of Parkinson’s disease and its driving factors in 195 countries and territories to 2050: modelling study of Global Burden of Disease Study 2021. BMJ.

[3] Kalia L.V., Lang A.E. (2015). Parkinson’s disease. Lancet.

[4] Tolosa E., Garrido A., Scholz S.W., Poewe W. (2021). Challenges in the diagnosis of Parkinson’s disease. Lancet Neurol.

[5] Li M., Ye X., Huang Z., Ye L., Chen C. (2025). Global burden of Parkinson's disease from 1990 to 2021: a population-based study. BMJ Open.

[6] GBD 2016 Parkinson’s Disease Collaborators (2018). Global, regional, and national burden of Parkinson’s disease, 1990–2016: a systematic analysis for the Global Burden of Disease Study 2016. Lancet Neurol.

[7] Zheng Z., Zhu Z., Zhou C., Cao L., Zhao G. (2023). Burden of Parkinson disease in China, 1990–2019: findings from the 2019 Global Burden of Disease Study. Neuroepidemiology.

[8] Xu T., Dong W., Liu J., Yin P., Wang Z., Zhang L. (2024). Disease burden of Parkinson’s disease in China and its provinces from 1990 to 2021: findings from the global burden of disease study 2021. Lancet Reg Health West Pac.

[9] Meng D., Wu J., Huang X., Liang X., Fang B. (2024). Prevalence of Parkinson’s disease among adults aged 45 years and older in China: a cross-sectional study based on the China health and retirement longitudinal study. BMC Public Health.

[10] Song Z., Liu S., Li X., Zhang M., Wang X., Shi Z. (2022). Prevalence of Parkinson’s disease in adults aged 65 years and older in China: a multicenter population-based survey. Neuroepidemiology.

[11] GBD 2019 Dementia Forecasting Collaborators (2022). Estimation of the global prevalence of dementia in 2019 and forecasted prevalence in 2050: an analysis for the Global Burden of Disease Study 2019. Lancet Public Health.

[12] GBD 2023 Disease and Injury and Risk Factor Collaborators (2025). Burden of 375 diseases and injuries, risk-attributable burden of 88 risk factors, and healthy life expectancy in 204 countries and territories, including 660 subnational locations, 1990–2023: a systematic analysis for the Global Burden of Disease Study 2023. Lancet.

[13] Global, regional, and national disability-adjusted life-years (DALYs) for 333 diseases and injuries and healthy life expectancy (HALE) for 195 countries and territories, 1990–2016: a systematic analysis for the Global Burden of Disease Study 2016. Lancet. 2017;390(10100):1260-1344. 10.1016/s0140-6736(17)32130-x PMC5605707 28919118

[14] Kim H.J., Fay M.P., Feuer E.J., Midthune D.N. (2000). Permutation tests for joinpoint regression with applications to cancer rates. Stat Med.

[15] Zhang C., Yang X., Wan D., Ma Q., Yin P., Zhou M. (2025). Burden of neurological disorders in China and its provinces, 1990–2021: findings from the global burden of disease study 2021. Med.

[16] Kontis V., Bennett J.E., Mathers C.D., Li G., Foreman K., Ezzati M. (2017). Future life expectancy in 35 industrialised countries: projections with a Bayesian model ensemble. Lancet.

[17] Reddy A., Reddy R.P., Roghani A.K., Garcia R.I., Khemka S., Pattoor V. (2024). Artificial intelligence in Parkinson’s disease: early detection and diagnostic advancements. Ageing Res Rev.

[18] Rutledge J., Lehallier B., Zarifkar P., Losada P.M., Shahid-Besanti M., Western D. (2024). Comprehensive proteomics of CSF, plasma, and urine identify DDC and other biomarkers of early Parkinson’s disease. Acta Neuropathol.

[19] Li G., Ma J., Cui S., He Y., Xiao Q., Liu J. (2019). Parkinson’s disease in China: a forty-year growing track of bedside work. Transl Neurodegener.

[20] Zhang Y., Wang C., Wang Y., Xiao Q., Liu J., Ma J. (2018). Mortality from Parkinson’s disease in China: findings from a ten-year follow up study in Shanghai. Parkinsonism Relat Disord.

[21] Gao F., Cheng X., Liu J., Han Y., Mao C., Zhong C. (2025). Incidence, mortality, and burden of Parkinson’s disease in China: a time-trend analysis and comparison with the global burden based on Global Burden of Disease Study 2021. Chin Med J (Engl).

[22] Xu W., Wang J., Li X.N., Liang J., Song L., Wu Y. (2023). Neuronal and synaptic adaptations underlying the benefits of deep brain stimulation for Parkinson's disease. Transl Neurodegener.

[23] Liu K., Hou T., Li Y., Tian N., Ren Y., Liu C. (2025). Development and internal validation of a risk prediction model for dementia in a rural older population in China. Alzheimers Dement.

[24] Cerri S., Mus L., Blandini F. (2019). Parkinson’s disease in women and men: what’s the difference?. J Parkinsons Dis.

[25] Cattaneo C., Pagonabarraga J. (2025). Sex differences in Parkinson’s disease: a narrative review. Neurol Ther.

[26] Templeton H.N., Ehrlich A.T., Schwerdtfeger L.A., Sheng J.A., Tjalkens R.B., Tobet S.A. (2025). Sex specific effects of environmental toxin-derived alpha synuclein on enteric neuronal-epithelial interactions. Neurogastroenterol Motil.

[27] GBD 2021 Tobacco Forecasting Collaborators (2024). Forecasting the effects of smoking prevalence scenarios on years of life lost and life expectancy from 2022 to 2050: a systematic analysis for the Global Burden of Disease Study 2021. Lancet Public Health.

[28] Kacprzyk K.W., Milewska M., Zarnowska A., Panczyk M., Rokicka G., Szostak-Wegierek D. (2022). Prevalence of malnutrition in patients with Parkinson’s disease: a systematic review. Nutrients.

[29] Santos-García D., de Deus Fonticoba T., Cores Bartolomé C., Feal Painceiras M.J., Íñiguez-Alvarado M.C., García Díaz I. (2023). Staging Parkinson’s disease according to the MNCD (motor/non-motor/cognition/dependency) classification correlates with disease severity and quality of life. J Parkinsons Dis.

[30] Ou Z., Pan J., Tang S., Duan D., Yu D., Nong H. (2021). Global trends in the incidence, prevalence, and years lived with disability of Parkinson’s disease in 204 countries/territories from 1990 to 2019. Front Public Health.

[31] Xu L., Wang Z., Li Q. (2025). Global trends and projections of Parkinson’s disease incidence: a 30-year analysis using GBD 2021 data. J Neurol.

[32] Tanner C.M., Kamel F., Ross G.W., Hoppin J.A., Goldman S.M., Korell M. (2011). Rotenone, paraquat, and Parkinson’s disease. Environ Health Perspect.

[33] Kanthasamy A., Jin H., Charli A., Vellareddy A., Kanthasamy A. (2019). Environmental neurotoxicant-induced dopaminergic neurodegeneration: a potential link to impaired neuroinflammatory mechanisms. Pharmacol Ther.

[34] De Miranda B.R., Goldman S.M., Miller G.W., Greenamyre J.T., Dorsey E.R. (2022). Preventing Parkinson’s disease: an environmental agenda. J Parkinsons Dis.

[35] Beach T.G., Adler C.H., Shill H.A., Zhang N., Driver-Dunckley E.D., Mehta S.H. (2023). Accuracy of the early diagnosis of Parkinson’s disease. Mov Disord.

[36] Höglinger G.U., Rissling I., Metz A., Ries V., Heinermann A., Prinz H. (2004). Enhancing recognition of early Parkinsonism in the community. Mov Disord.

[37] Wang A., Costello S., Cockburn M., Zhang X., Bronstein J., Ritz B. (2011). Parkinson’s disease risk from ambient exposure to pesticides. Eur J Epidemiol.

[38] Wang W., Wu Q., Yang X., He X., An X. (2017). Migrant elders in Hainan, China, report better health and lower depression than local-born elderly residents. Ann Hum Biol.

[39] Okun M.S. (2012). Deep-brain stimulation for Parkinson’s disease. N Engl J Med.

[40] Tabar V., Sarva H., Lozano A.M., Fasano A., Kalia S.K., Yu K.K.H. (2025). Phase I trial of hES cell-derived dopaminergic neurons for Parkinson’s disease. Nature.

[41] Zhuo Y., Li W.S., Lu W., Li X., Ge L.T., Huang Y. (2024). TGF-β1 mediates hypoxia-preconditioned olfactory mucosa mesenchymal stem cells improved neural functional recovery in Parkinson’s disease models and patients. Mil Med Res.

