# Supplementary material for: Trends and future projections of Parkinson’s disease burden in China: findings from the Global Burden of Disease Study 2023
Source: Mil Med Res. 2026 Oct 27;13(1):100077. doi: 10.1016/j.mmr.2026.100077 (PMC13734925; doi:10.1016/j.mmr.2026.100077)
Supplement: Supplementary file 1 [file mmc1.pdf]

Methods

Age-standardized rate (ASRs) calculation

ASRs were calculated using the direct standardization method to account for differences in age structure across populations and over time. For each year, province, and sex, age-specific rates were first computed for predefined age groups. These rates were then weighted using the age distribution of the GBD 2023 global standard population.

Formally, the ASR was calculated as:

$$\text{ASR} = \frac{\sum_a r_a \times w_a}{\sum_a w_a} \times 100{,}000$$

where r_a denotes the age-specific rate for age group a, and w_a represents the corresponding age-group weight from the GBD 2023 standard population. Rates were expressed per 100,000 population. Uncertainty intervals (UIs) for ASRs were propagated by applying the same standardization procedure to each draw of the posterior distribution provided by GBD 2023 and summarizing the resulting distributions using the 2.5th and 97.5th percentiles.

Temporal trend analysis

Long-term temporal trends were characterized using joinpoint regression applied to log-transformed outcome series, allowing up to three joinpoints. The maximum number of joinpoints was set to three because the study period included 34 annual observations from 1990 to 2023. This setting allowed the model to capture major long-term changes in temporal trends while avoiding excessive segmentation of the time series. In addition, each segment was required to contain at least eight observations, which further reduced the risk of fitting short-term fluctuations. The final number of joinpoints was not fixed a priori but was selected from candidate models with zero to three joinpoints using the Bayesian Information Criterion (BIC), thereby balancing model flexibility against overfitting. For each segment j, the estimated annual percentage change (EAPC) was calculated as:

$$\text{EAPC}_j = 100 \times \left(\exp(\beta_j) - 1\right)$$

where β_j denotes the slope coefficient of segment j on the log scale.

The average annual percentage change (AAPC) over the full study period was computed as a time-

weighted average of the segment-specific EAPCs:

$$\text{AAPC} = \frac{\sum_{j=1}^{J} w_j \times \text{EAPC}_j}{\sum_{j=1}^{J} w_j}$$

where J is the total number of segments and w_j represents the length (in years) of segment j. CIs for the AAPC were derived from the fitted joinpoint model using the delta method, based on the variance–covariance matrix of the segment-specific slope estimates, and reported as 95% confidence intervals (CIs).

Decomposition analysis of changes in PD cases

We quantified the relative contributions of population growth, population ageing, and changes in age-specific rates to the observed change in PD cases using a Das Gupta-style decomposition. For each province and sex (Both), we extracted age-specific PD numbers and rates for the baseline and comparison years, excluding “All ages” and “Age-standardized” categories. Age-specific population was derived as:

$$P_a = \frac{\text{Number}_a}{\text{Rate}_a} \times 100{,}000$$

where P_a denotes the population in age group a, Number_a is the number of PD cases, and Rate_a is the corresponding age-specific rate. Total PD cases were expressed as:

$$C = \sum_{a} \frac{P_a r_a}{100{,}000}$$

where r_a represents the age-specific rate. Equivalently, this can be written as:

$$C = P \sum_{a} \frac{s_a r_a}{100{,}000}$$

where $P = \sum_a P_a$ is the total population and $s_a = P_a/P$ denotes the population share of age group a.

The total change in PD cases between the two points was decomposed into three components: i) the population growth effect (changes in total population size), ii) the population ageing effect (changes in age structure), and iii) the age-specific rate effect (changes in disease risk within age groups). Each component was expressed as a percentage of baseline cases.

Forecasting of PD burden to 2050

We projected PD burden through 2050 using an ensemble probabilistic model-averaging framework [2], which was fitted to population-level annual time-series data from the 2023 GBD, rather than an individual-

level etiological or mortality risk model. Medications, comorbidities, and environmental or behavioral exposures were not included as explicit predictors because consistent longitudinal covariate data were unavailable at the required outcome-specific and geographical resolution. Forecasts were generated for the absolute numbers and ASRs of PD prevalence, incidence, deaths, DALYs, YLLs, and YLDs, where applicable. To stabilize variance and ensure non-negativity, each time series (y_t) was analyzed on the logarithmic scale and defined as

$$y_t = \log(\max(x_t, \varepsilon))$$

where x_t denotes the observed outcome in year t, and ε is a small positive constant used when $x_t = 0$.

A set of candidate trend models was fitted to each transformed series, including linear and quadratic regressions on the calendar year, natural cubic spline regression with fixed degrees of freedom, piecewise linear regression with a single unknown knot selected by minimizing the Bayesian Information Criterion (BIC), an automatically selected autoregressive integrated moving average (ARIMA) model, and a random-walk model specified as ARIMA (0,1,0). For each regression-based model, we additionally constructed a residual-corrected specification by modeling the regression residuals using a random-walk process and adding the forecasted residual component back to the fitted trend, thereby capturing the temporal dependence not explained by the deterministic mean structure.

To limit implausible long-term extrapolation, the residual random-walk component was damped and soft caps were applied based on the range observed in the most recent period, constraining extreme forecasts while preserving short-term dynamics. Model performance was evaluated using an out-of-sample validation strategy in which the most recent 11 years of data were withheld, models were fitted to the earlier period, and forecasts were compared against the withheld observations using the root mean squared error (RMSE) and bias. Because model fitting was performed on a log scale, the predicted values were back-transformed to the original scale before validation. The RMSE was used to quantify the overall magnitude of the prediction error, whereas bias was used to assess systematic over- or under-prediction. These validation metrics were used to examine the stability and plausibility of the candidate models before

generating the final projections for 2050.

Model weights were derived using BIC-based model averaging. For residual-corrected models, composite BIC values were approximated as the sum of the BIC values from the core regression model and the residual model. We first define

$$\mathrm{BIC}_{\min} = \min_{k} \mathrm{BIC}_k$$

and the weight assigned to the model is calculated as w_m

$$w_m = \frac{\exp\left[-\frac{1}{2}(\mathrm{BIC}_m - \mathrm{BIC}_{\min})\right]}{\sum_k \exp\left[-\frac{1}{2}(\mathrm{BIC}_k - \mathrm{BIC}_{\min})\right]}$$

To quantify forecasting uncertainty, model-based posterior predictive simulations were generated for each candidate model. For regression-based models, coefficients were sampled from their estimated multivariate normal distributions, whereas for the time-series models, future innovations were simulated. Where applicable, residual random walk components were also simulated. The final predictive distribution for each forecast year t is obtained by combining the model-specific predictive distributions according to their BIC-based model weights as follows $p(\tilde{y}_t)$.

$$p(\tilde{y}_t) = \sum_{m} w_m \, p_m(\tilde{y}_t)$$

where $p_m(\tilde{y}_t)$ denotes the predictive distribution for forecast year t under model m, w_m is the corresponding BIC-based model weight. Final forecasts were summarized as the mean of the combined predictive distribution, with 95% UIs defined by the 2.5th and 97.5th percentiles.

Figure legends

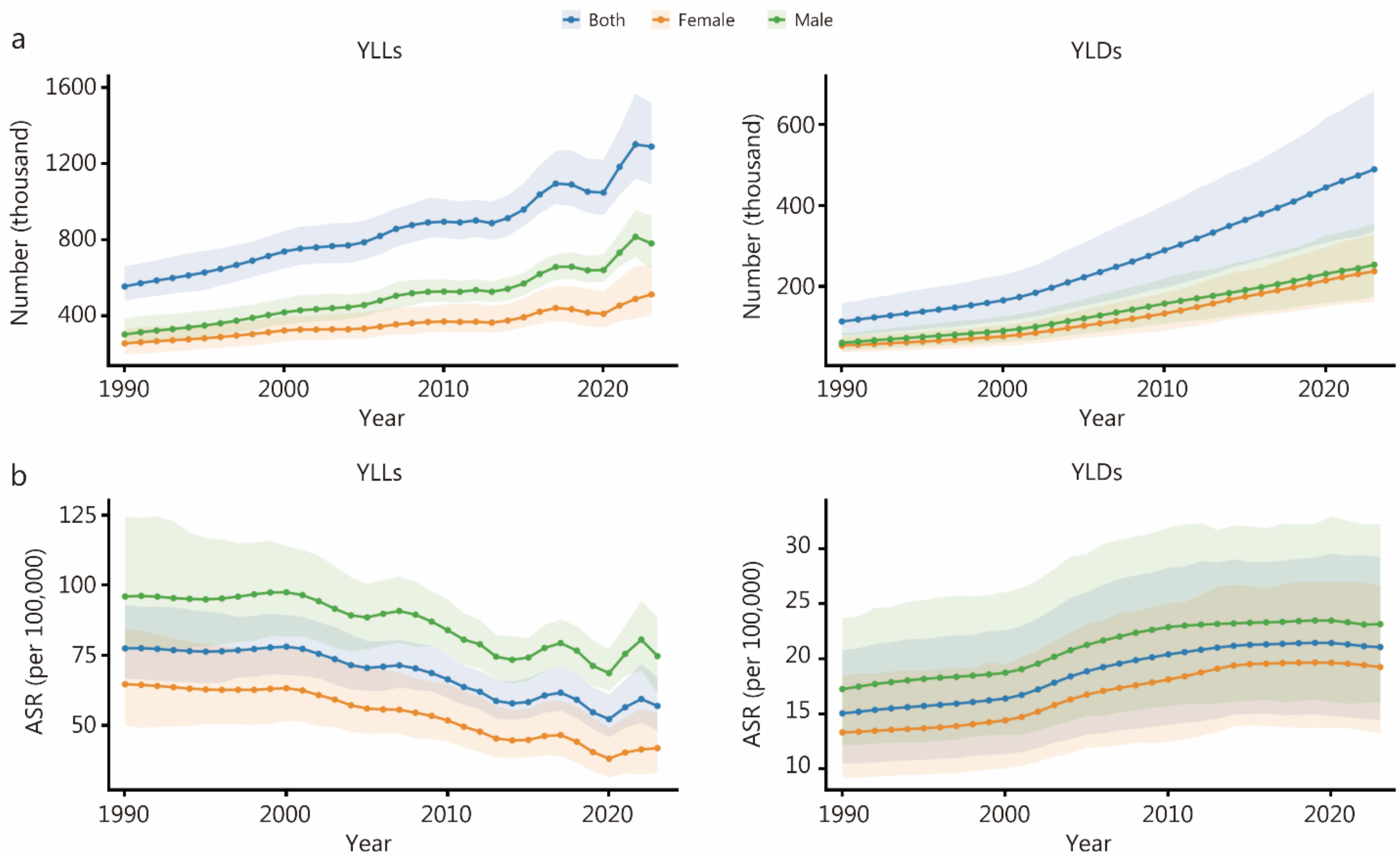

Fig. S1 Annual trends in YLLs and YLDs from PD by sex in China, 1990–2023. **a** Number of YLLs and YLDs. **b** Age-standardized rate (ASR) of YLLs and YLDs. Estimates are shown for both sexes combined, females, and males. Numbers are expressed in thousands, and ASRs are expressed per 100,000. Shaded areas denote 95% uncertainty intervals (UI). PD. Parkinson’s disease; YLLs. Years of life lost; YLDs. Years lived with disability

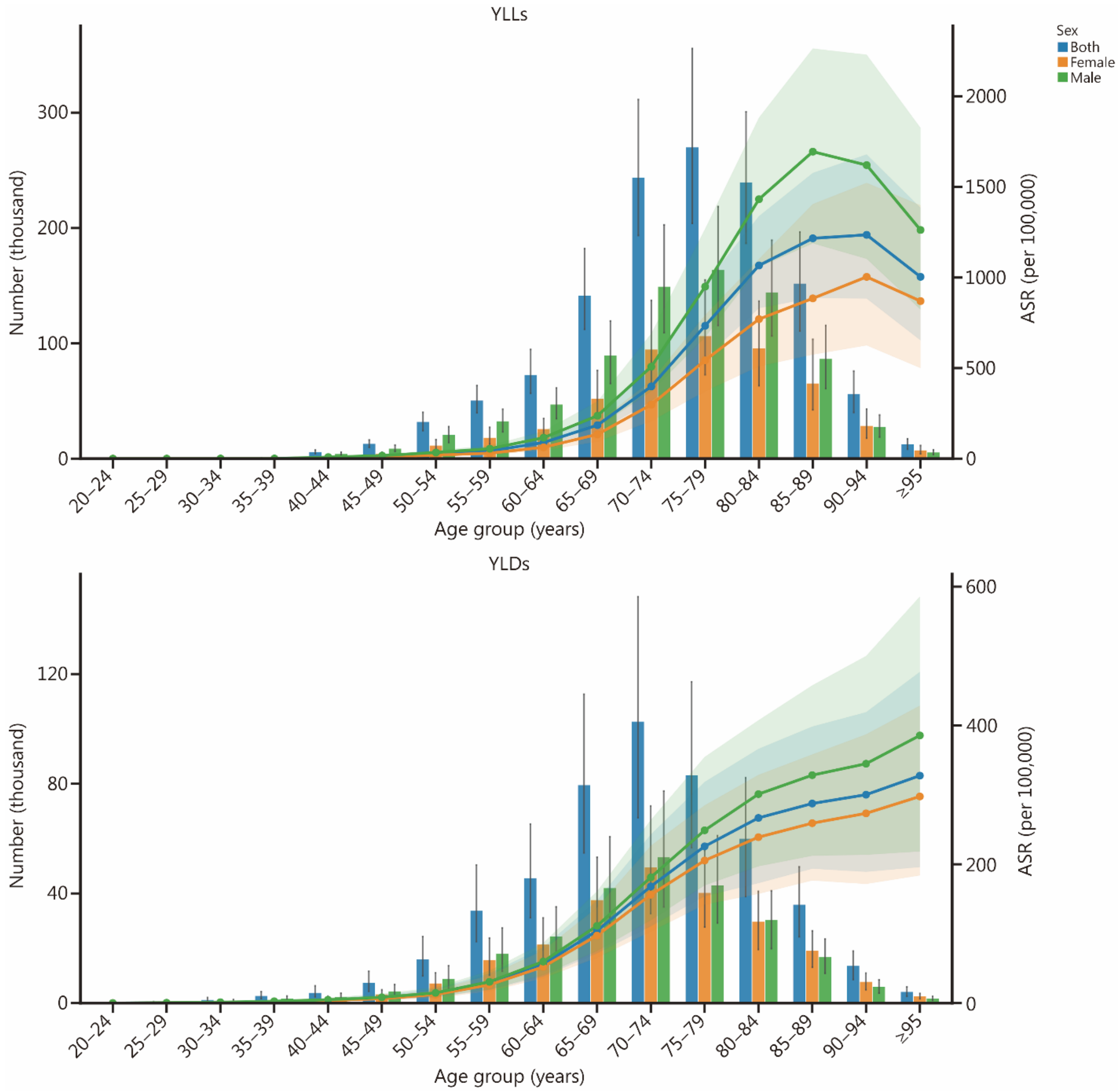

Fig. S2 Age-specific YLL and YLD burden of PD by sex in China, 2023. Bars represent absolute numbers, expressed in thousands, and lines represent the corresponding age-specific rates per 100,000 for both sexes combined, females, and males. Error bars and shaded areas indicate 95% uncertainty intervals (UIs). PD. Parkinson’s disease; YLLs. Years of life lost; YLDs. Years lived with disability

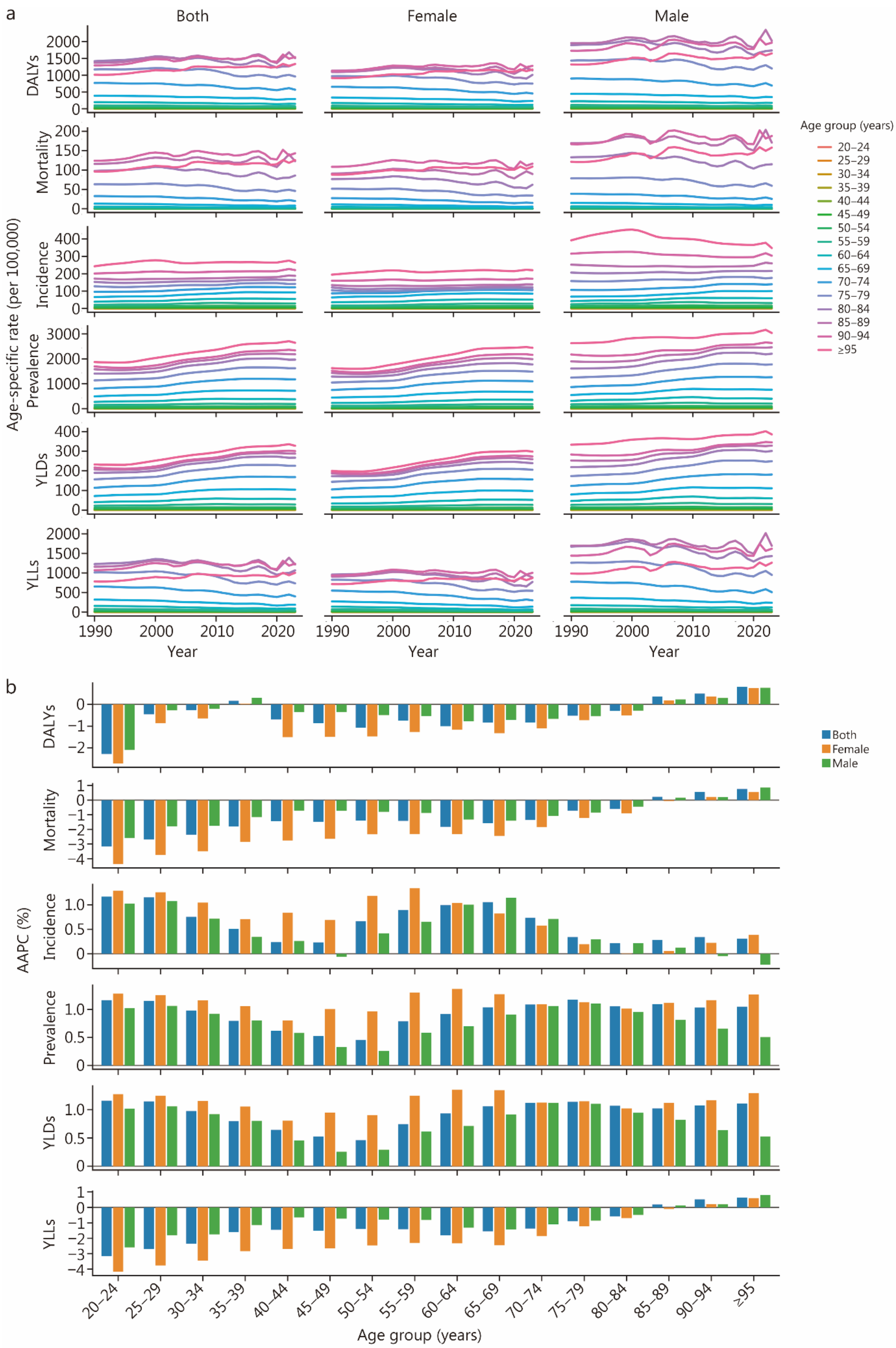

Fig. S3 Temporal trends and average annual percentage changes in age-specific PD burden by age group and sex in China, 1990–2023. **a** Temporal trend in age-specific rates of DALYs, mortality, incidence, prevalence, YLDs, and YLLs from 1990 to 2023, stratified by age group and sex. **b** AAPCs in the corresponding rates from 1990 to 2023 across age groups, shown for both sexes combined, females, and males. PD. Parkinson's disease; DALYs. Disability-adjusted life years; YLDs. Years lived with disability; YLLs. Years of life lost; AAPC. Average annual percentage change

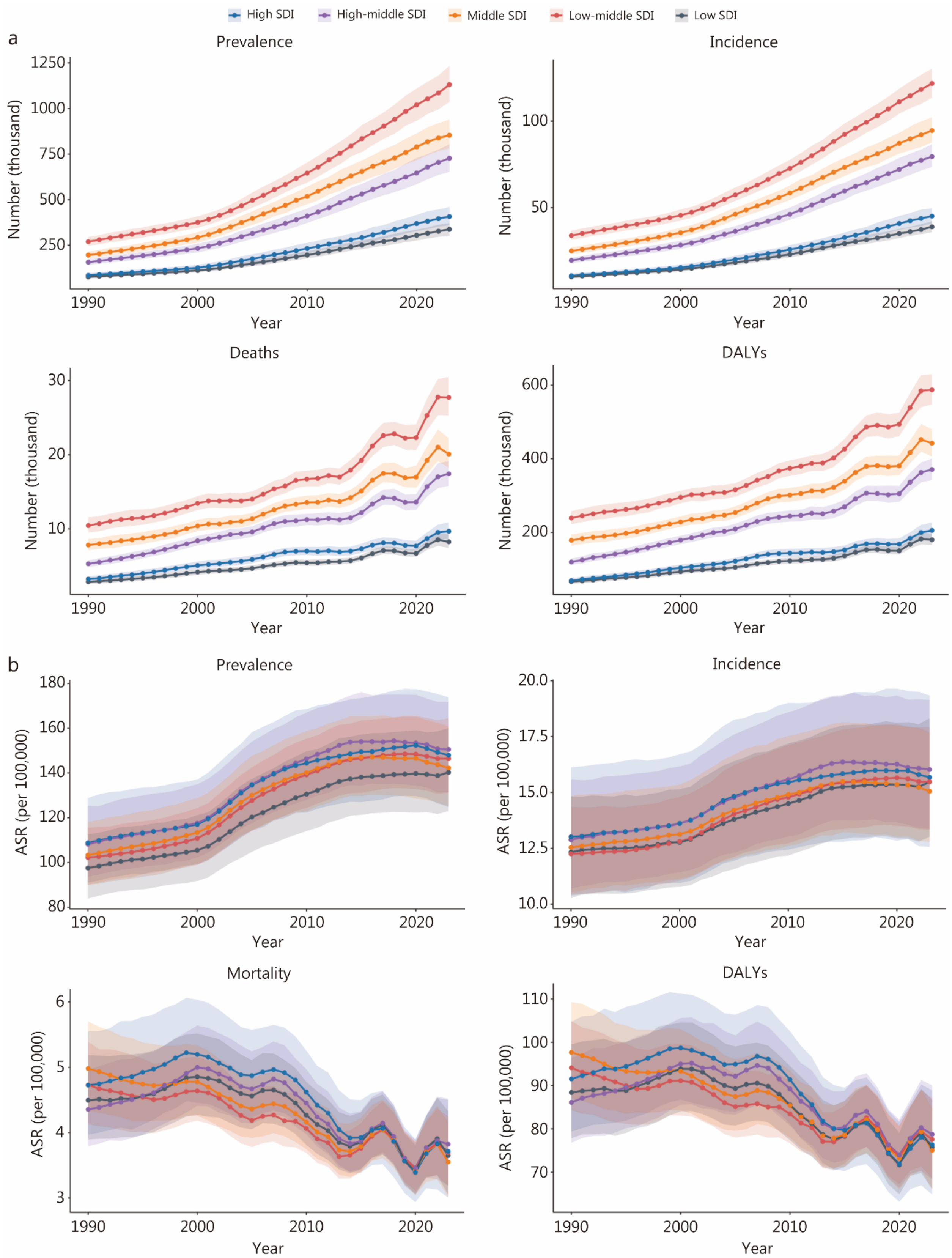

Fig. S4 Temporal trends in PD prevalence, incidence, deaths, and DALYs by SDI group in China, 1990–2023. Numbers of prevalence, incidence, deaths, and DALYs (**a**) are expressed in thousands; age-standardized rate (ASR), including prevalence, incidence, deaths, and DALYs (**b**) are expressed per 100,000. Estimates are shown for high-, high-middle-, middle-, low-middle-, and low-SDI groups. Shaded areas indicate 95% uncertainty intervals (UI). PD. Parkinson's disease; DALYs. Disability-adjusted life years; SDI. Socio-demographic Index

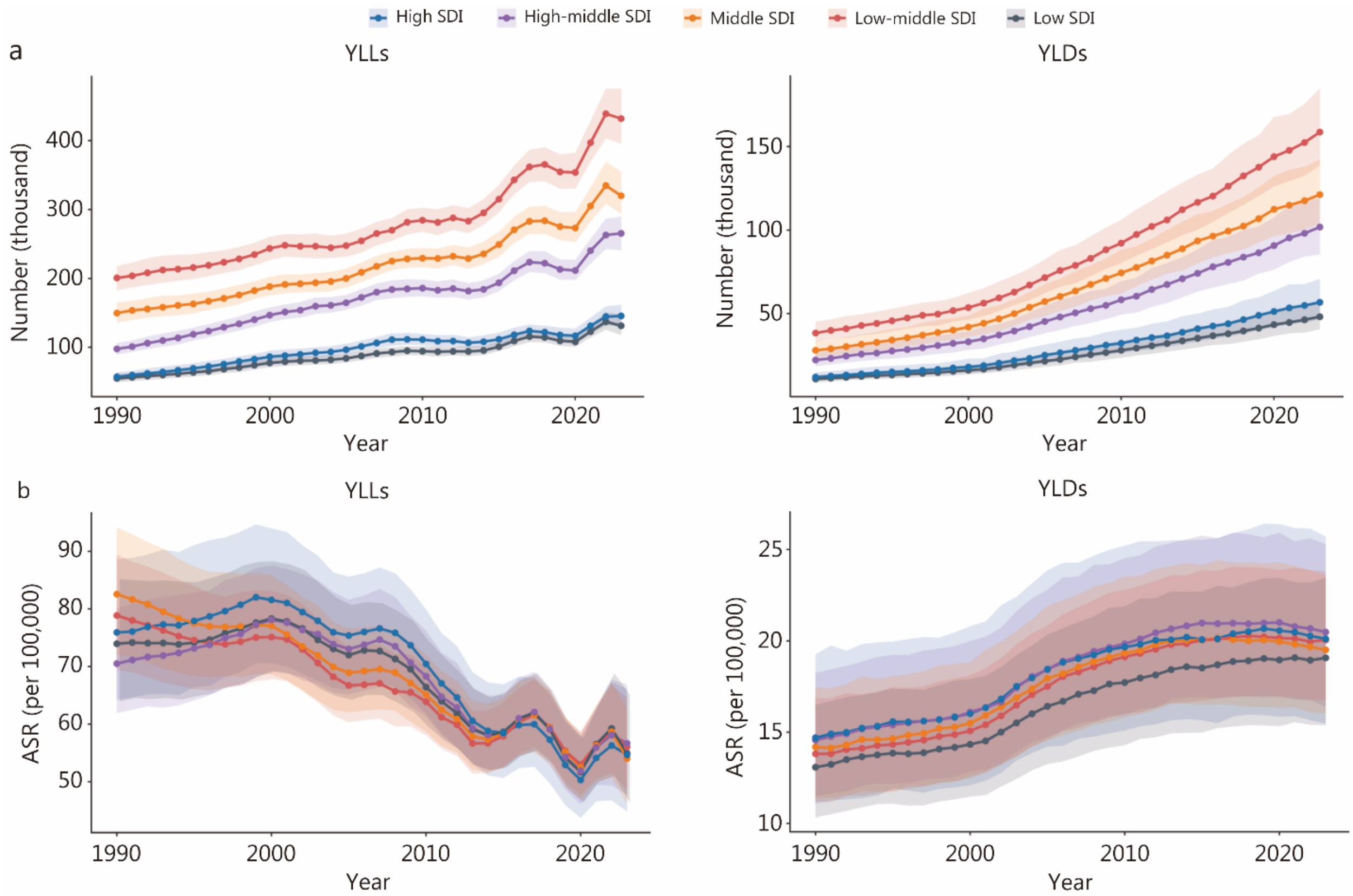

Fig. S5 Temporal trends in PD-related YLLs and YLDs by SDI group in China, 1990–2023. **a** Number of YLLs and YLDs; **b** Age-standardized rate (ASR) of YLLs and YLDs. Estimates are shown for high-, high-middle-, middle-, low-middle-, and low-SDI groups. Shaded areas indicate 95% uncertainty intervals (UI). PD. Parkinson's disease; YLLs. Years of life lost; YLDs. Years lived with disability; SDI. Socio-demographic Index

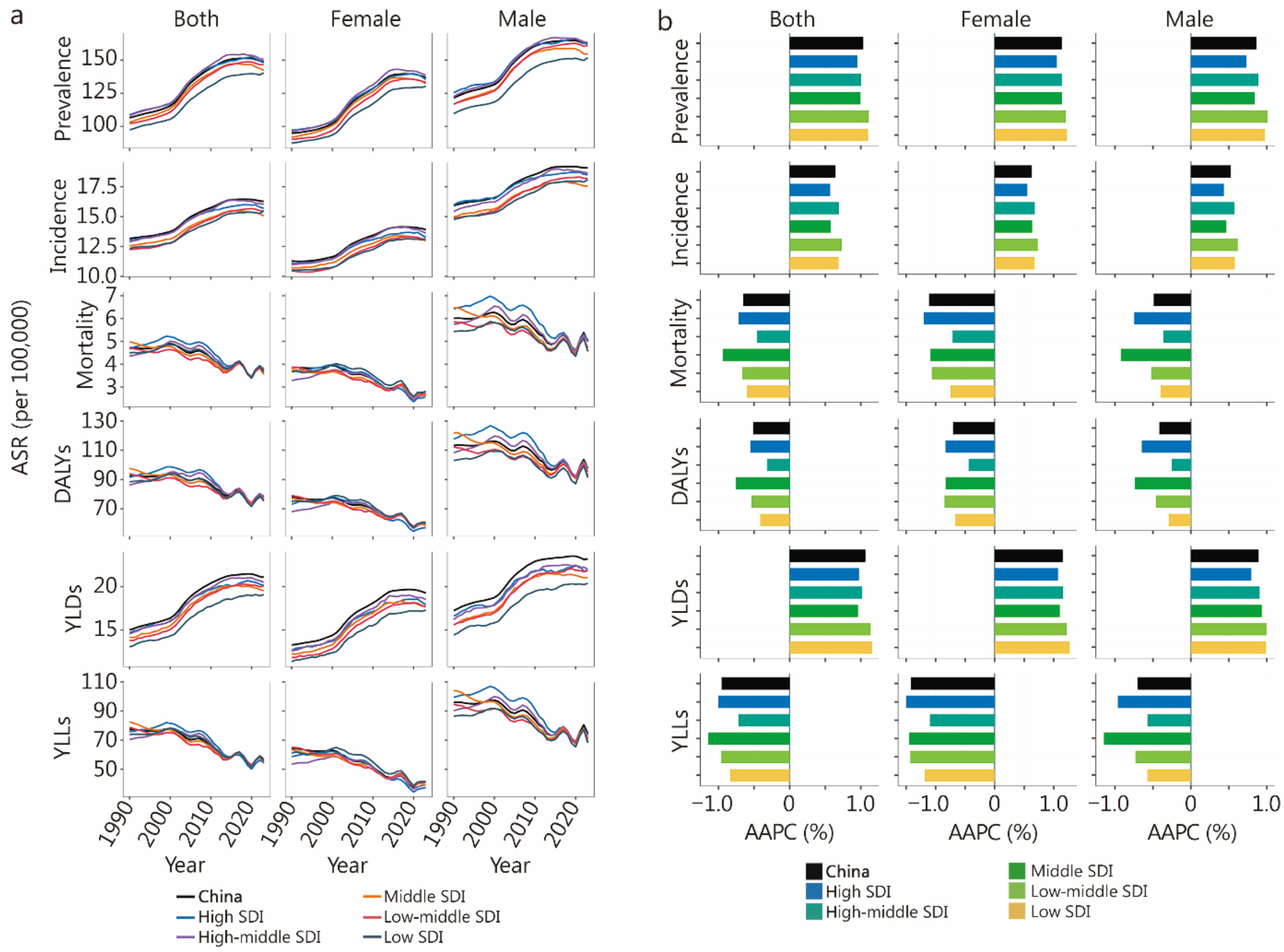

Fig. S6 Temporal trends in age-standardized rates (ASRs) and corresponding AAPCs of PD burden by SDI group and sex in China, 1990–2023. **a** ASRs of prevalence, incidence, mortality, DALYs, YLDs, and YLLs for China overall and across five SDI groups, stratified by sex. **b** Corresponding AAPCs in these ASRs from 1990 to 2023 for both sexes combined, females, and males. Positive AAPC values indicate increasing trends, whereas negative values indicate decreasing trends. PD. Parkinson's disease; AAPC. Average annual percentage change; SDI. Socio-demographic Index; DALYs. Disability-adjusted life years; YLDs. Years lived with disability; YLLs. Years of life lost

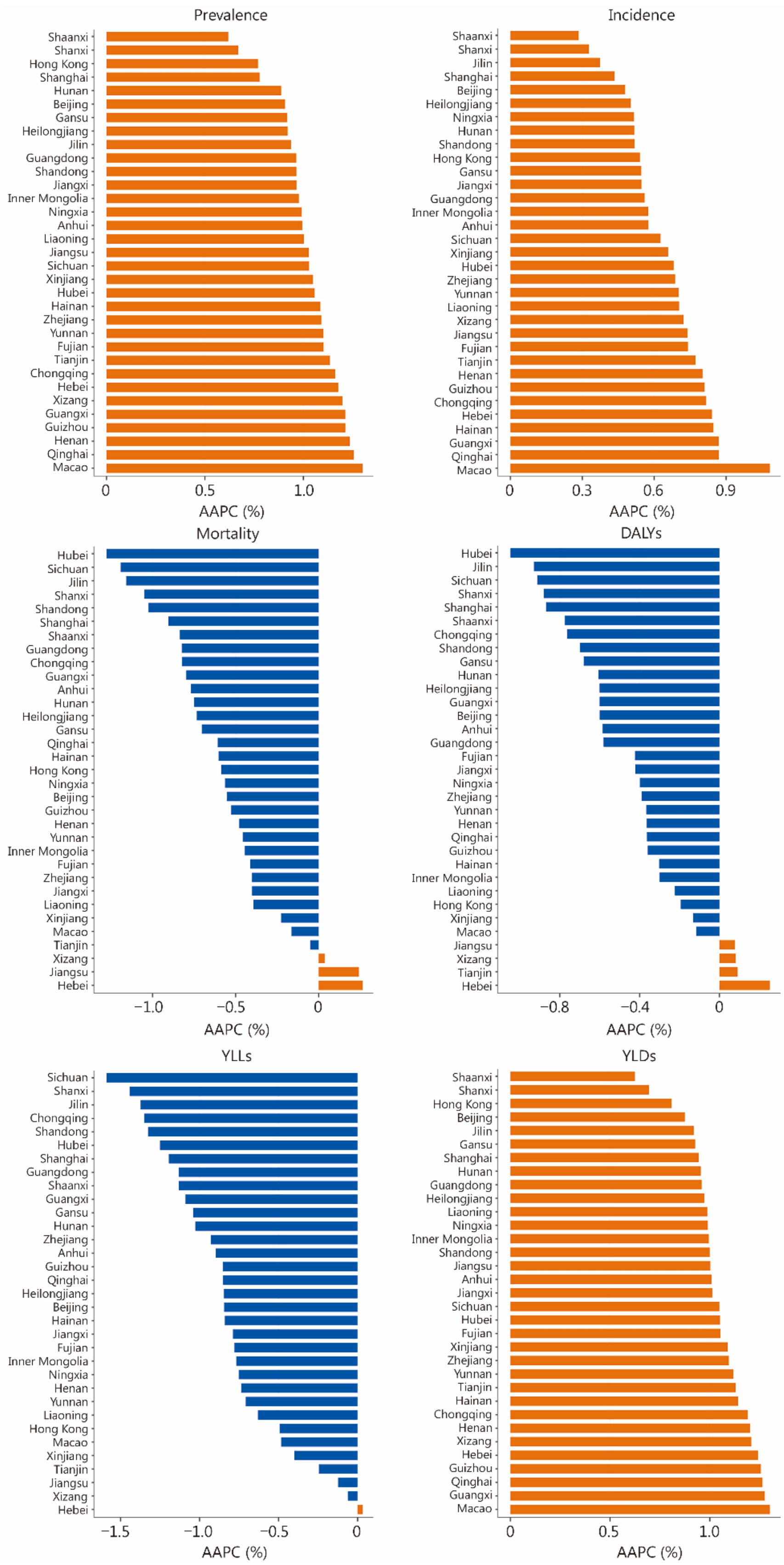

Fig. S7 Provincial variation in Average annual percentage changes (AAPCs) of age-standardized PD burden rates in China, 1990–2023. AAPCs in the age-standardized prevalence, incidence, mortality, DALYs, YLLs, and YLDs rates across China. Positive AAPC values indicate increasing trends, whereas negative values indicate decreasing trends. PD. Parkinson's disease; AAPC. Average annual percentage change; DALYs. Disability-adjusted life years; YLLs. Years of life lost; YLDs. Years lived with disability

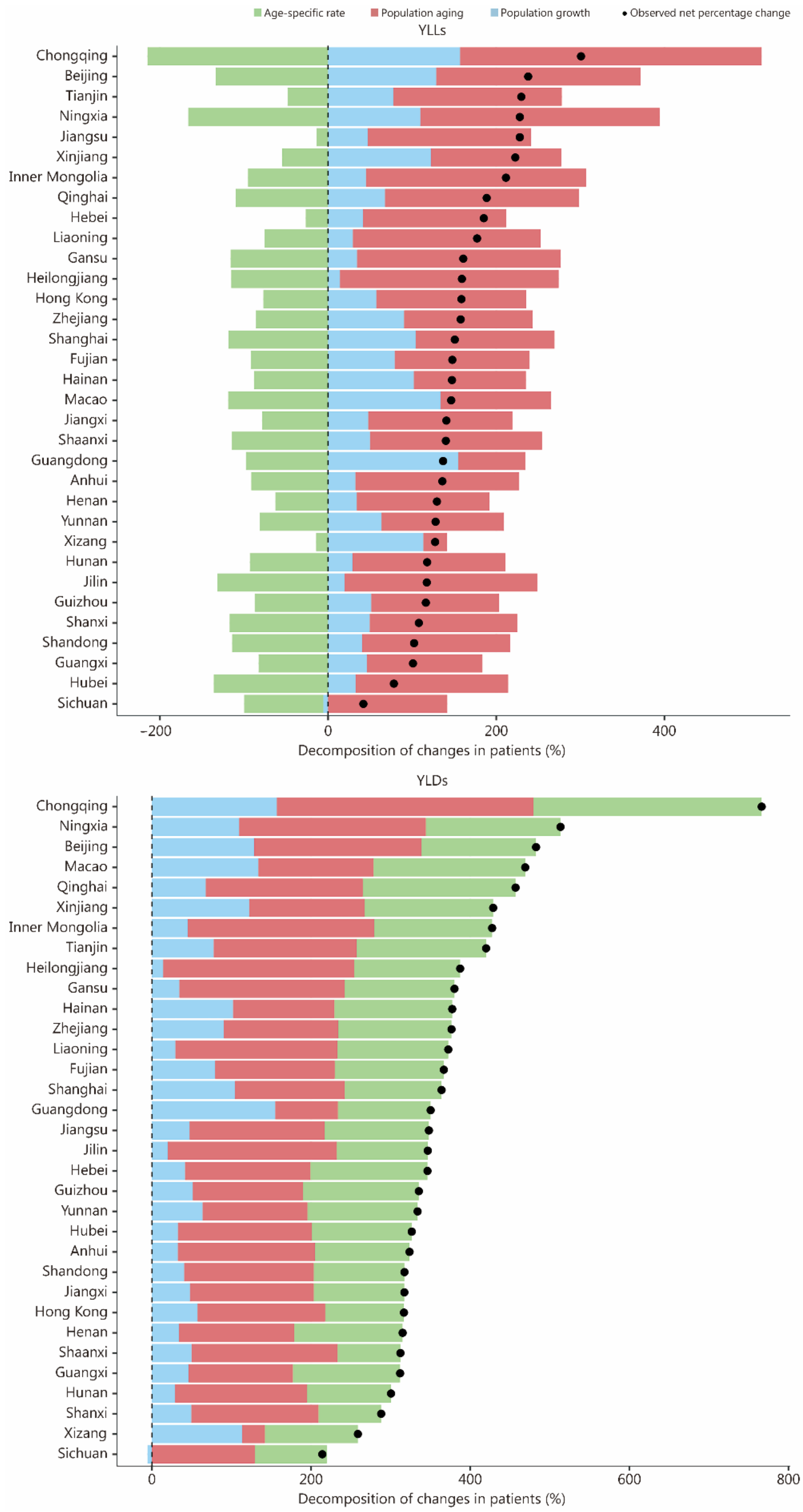

Fig. S8 Decomposition of changes in PD-related YLLs and YLDs across China, 1990–2023. Decomposition of changes in YLLs and YLDs into contributions from population growth, population aging, and changes in age-specific rates. Stacked bars show the contribution of each component, expressed as a percentage of the corresponding 1990 burden. Positive values indicate contributions to an increase in burden, whereas negative values indicate contributions to a decrease. Black dots denote the net percentage change in YLLs or YLDs between 1990 and 2023. PD. Parkinson's disease; YLLs. Years of life lost; YLDs. Years lived with disability

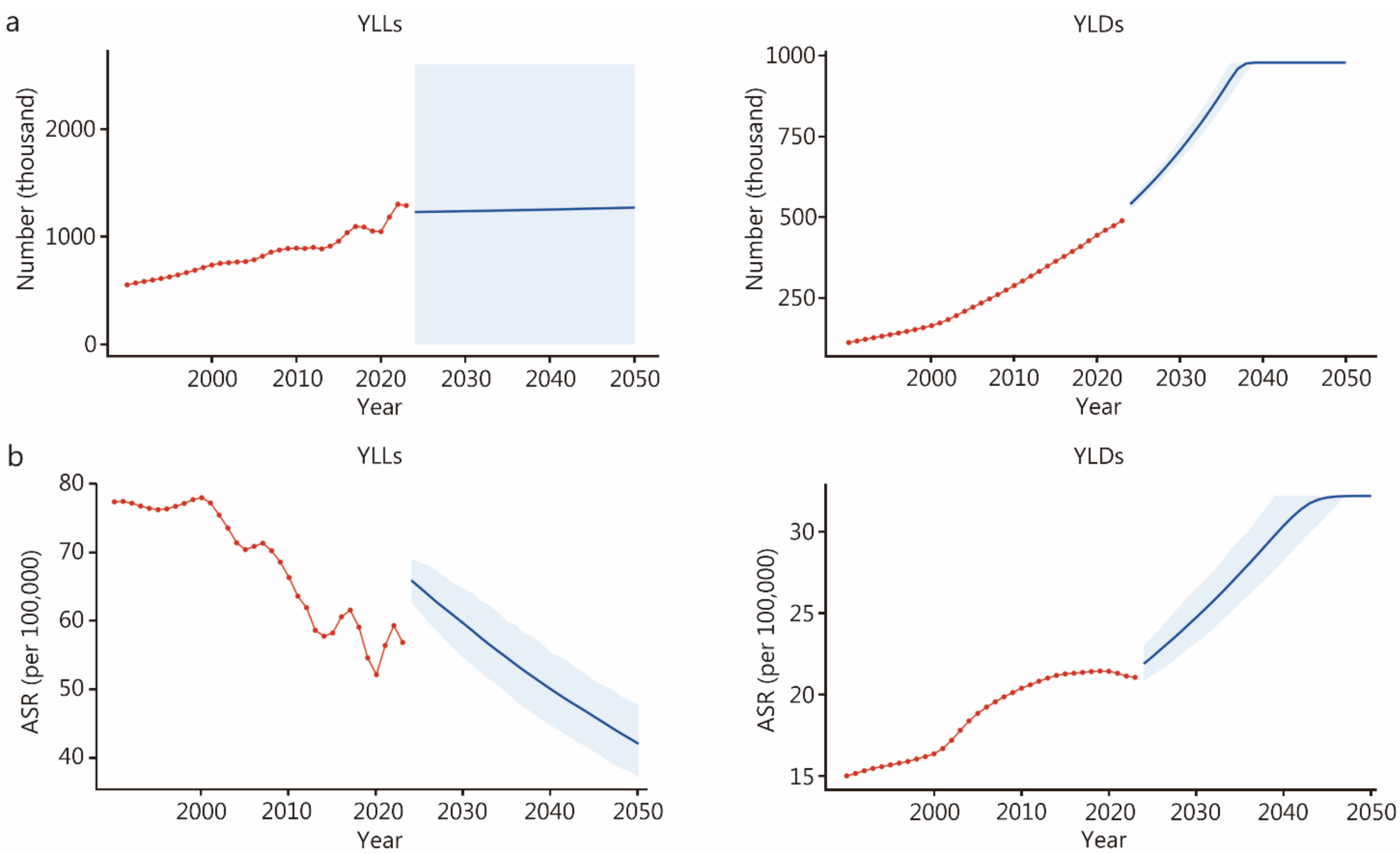

Fig. S9 Projected trends in PD-related YLLs and YLDs in China, 2024–2050. Observed and projected numbers of YLLs and YLDs (**a**), together with the corresponding age-standardized rates (ASRs) of YLLs and YLDs (**b**). Red lines and points represent observed estimates for 1990–2023, whereas blue lines represent projections for 2024–2050. Shaded areas denote 95% uncertainty intervals (UIs). Projections were generated using an ensemble probabilistic model-averaging framework. PD. Parkinson's disease; YLLs. Years of life lost; YLDs. Years lived with disability
